# Spin Balance Over Janus Ir-Co Magnetic Atoms for Efficient Acidic Water Oxidation

**DOI:** 10.1007/s40820-026-02082-2

**Published:** 2026-01-28

**Authors:** Na Li, Weiren Cheng, Yuying Liu, Ruiqi Liu, Sihua Feng, Huijuan Wang, Liyang Lv, Chenglong Liu, Jin Ma, Chao Wang, Wensheng Yan

**Affiliations:** 1 National Synchrotron Radiation Laboratory, University of Science and Technology of China, Hefei, Anhui 230029 People’s Republic of China; 2 Key Laboratory of Precision and Intelligent Chemistry, Department of Materials Science and Engineering, University of Science and Technology of China, Hefei, Anhui 230026 People’s Republic of China; 3 Material Test and Analysis Lab, Energy and Materials Science Experiment Center, University of Science and Technology of China, Hefei, Anhui 230029 People’s Republic of China

**Keywords:** Spin state, Superoxide path mechanism, Intermediate spin state, O *K* edge adsorption spectroscopy, In situ Raman

## Abstract

Herein, spin regulation of the low-spin Ir^4+^ in Ca_2_IrO_4_ is realized via a novel spin balance strategy through a Janus Ir–Co structure using high-spin Co^3+^ dopants, achieving the intermediate-spin state of Ir and Co atoms and enhancing the acidic oxygen evolution reaction (OER) performance of the obtained catalysts (Co–CIO). The optimized 0.2Co–CIO catalyst, with a nominal Co/(Co + Ir) metal atom percentage of 20%, displays exceptional electrochemical water oxidation activity with an ultrasmall overpotential of ~ 200 mV at 10 mA cm^−2^, ultralarge mass activity of 1110 A g_Ir_^−1^, and high turnover frequency of 2050 h^‒1^ under an overpotential of 300 mV in 1 M HClO_4_, outperforming most recently reported Ir–based oxides catalysts. Molecular and atomic characterizations via in situ X-ray absorption near-edge and Raman spectroscopy demonstrate the acceleration of bridged O‒O formation over the Janus Ir–Co units, indicating a preference for the superoxide path mechanism for Co–CIO. Furthermore, density functional theory calculations rationalize the promotion of the superoxide *O‒O intermediate over the spin-regulated Ir‒O‒Co units, thanks to optimized *e*_g_^1^ orbital and reduced *t*_2g_ orbital occupancy. The study presents a rare example of Ir spin regulation via a Janus Ir–Co magnetic structure, thereby promoting acidic OER activity.

**Supplementary Information:**

The online version contains supplementary material available at 10.1007/s40820-026-02082-2.

## Highlights

- Monodisperse and substitute Co were doped into edge-sharing [IrO_6_] octahedra of Ca_2_IrO_4_ model catalyst, which usually present the intrinsic and strong stability for acid oxygen evolution reaction (OER)
- The optimized Janus Co–Ir local structure triggers spin balance effect with optimal e_g_^1^ orbital and uneven t_2g_ orbital despite the large crystal field of Ir, which co-promote the OER activity with a relatively stable crystal structure.
- Different from the slowly kinetics of adsorbates evolution mechanism on Ca_2_IrO_4_, superoxide path mechanism occurs on Co doped Ca_2_IrO_4_ based on the assignment of *OO on dual active sites of Ir and Co.

**Supplementary Information:**

The online version contains supplementary material available at 10.1007/s40820-026-02082-2.

## Graphical abstract

**Figure:**
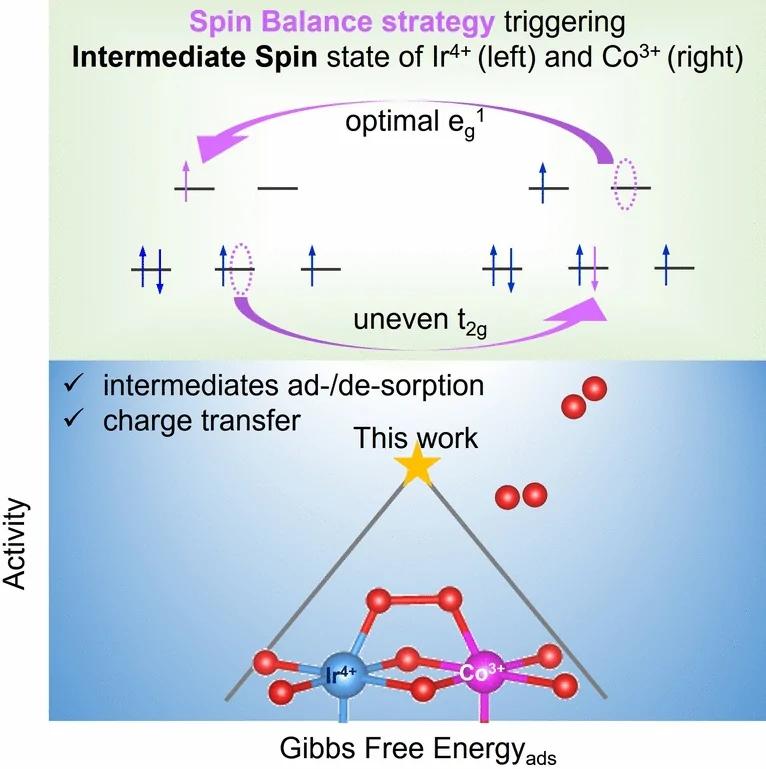

## Introduction

Hydrogen fuel is a promising candidate for the storage of renewable energy (e.g., wind and solar power) owing to its high energy density [1, 2]. Among various sustainable hydrogen generation systems, acidic water electrolysis exhibits faster hydrogen evolution reaction kinetics than its alkali counterpart [3]. However, its catalytic efficiency is mainly hampered by the complex four-electron transfer process during the oxygen evolution reaction (OER) at the electrolyzer anode. Although Ir-based oxides are state-of-the-art electrocatalysts for OER due to their lower overpotential and greater chemical stability under acidic conditions than other electrocatalysts [4–6], the scarcity and high cost of Ir limits their large-scale application. Thus, the development of affordable Ir-based OER catalysts with enhanced Ir mass activity and reduced Ir loading is essential.

Currently, a comprehensive understanding of OER activity should include the electron spin characteristics of electrocatalysts, which can not only reduce thermodynamic barriers during the adsorption/desorption of intermediates but also accelerate electron transfer reaction kinetics [7–10]. Previous experimental studies demonstrated the generation of key Ir = O or Ir–*O (↑↓) intermediates during the OER process [11, 12], wherein paired covalent electrons in π orbitals contributed to strong binding and poor activity. By contrast, the Ir–*O (↑↑) intermediate harbored electrons in parallel spin alignment [13] with optimal adsorption/desorption energy [14]. The electron transfer process is affected by successive spin polarizations along cation–anion–cation segments, wherein the valence electronic configuration of doped heteroatoms could induce changes in *t*_*2g*_ and/or *e*_*g*_ orbital filling, thereby optimizing OER performance. Experimentally, the key for spin-related OER enhancement is the changed spin state of active metal sites. Although previously reported for 3*d* transition metal like Co or Fe atoms [15, 16], spin change are rarely reported for Ir site due to the large crystal field splitting [17] and the lack of proper analysis method. For example, Gao et al. reported the changed d band center and bandwidth of Ir sites for the optimized binding of OER reaction intermediates through the introduction of higher magnetic moment of Fe atoms [18]. Therefore, to further boost OER activity of Ir-based oxides via desired valence orbitals and optimal electron arrangement, regulating and precisely analysis the spin state of Ir atoms remains a major challenge.

Perovskite-type complex oxides with lower Ir content, such as ABO_3_- and A_n+1_B_n_O_3n+1_-type oxides, are regarded as promising acidic OER electrocatalysts owing to their highly adjustable composition and electronic structures [19–25]. Additionally, their strong IrO_6_ octahedron structures, such as face-sharing dimers or edge-sharing one-dimensional (1D) chains, provide a highly stable basis for understanding the spin-dependent interactions between catalysts and intermediates in an acidic environment [12, 26]. We previously demonstrated the long durability of active Ca_2_IrO_4_ (CIO) lasts for 300 h at a current density of 10 mA cm^‒2^ in 1 M HClO_4_, which is due to the unique strong edge-sharing IrO_6_ octahedron structure [12]. Substitution with various non-noble metals at the Ir site has also been proposed to further reduce Ir loading while creating unique physicochemical properties to promote OER performance [27–30]. In this letter, we focus on the regulation of Ir spintronic states through inter-atom exchange interactions via the introduction of magnetic heteroatoms (M) to form Janus Ir–M structure. These constructed structure is helpful in systematic and comprehensive mechanism studies, especially under in situ conditions, for the future design and synthesis of more cost-effective OER catalysts.

Herein, we report spin balance over the Janus Ir–Co structure in Co-doped Ca_2_IrO_4_ catalysts (Co–CIO) for the first time, thereby achieving superior OER activity compared to previously reported Ir oxide–based electrocatalysts. Doping with high-spin (HS) Co atoms, the initial low-spin (LS) state of Ir atoms, with the electron configuration of *t*_2g_^5^*e*_g_^0^, can be reasonably optimized to intermediate-spin (IS) state, with a *t*_2g_^4^*e*_g_^1^ configuration, accompanied by the switching of HS to IS Co atoms. The reduced electron occupancy of the Ir 5*d t*_2g_ orbital effectively weakens electron repulsion in Ir *t*_2g_‒O p hybridization, while optimal *e*_g_^1^ orbital filling for the Ir and Co sites optimizes the intermediate-binding capacity to significantly improve electrocatalytic activity. Specifically, the 0.2Co–CIO catalyst exhibits a low overpotential of approximately 200 mV at a current density of 10 mA cm^‒2^, achieves an ultrahigh mass activity of 1110 A g_Ir_^‒1^, and demonstrates a high turnover frequency (TOF) of 2050 h^‒1^ at 1.53 V versus RHE, which is 140–190 times larger than that of commercial IrO_2_. In situ X-ray absorption near-edge spectroscopy (XANES) and Raman spectroscopy reveal the prompt generation of *O‒O species over the Janus Ir–Co structure during the OER process. The density functional theory (DFT) calculations confirm that the energy barrier of reaction-determining step (RDS) along conventional adsorption evolution mechanism (AEM) on single Ir sites is overcomed via boosting the superoxide path mechanism (SOPM) over the spin-regulated Ir‒O‒Co units in Co–CIO. The study findings suggest a new paradigm for the design of spin-related electrocatalysts and provide meaningful insight into the catalytic mechanism at the spintronic level.

## Experimental Section

### Materials

K_2_IrCl_6_ (99.99%) and IrO_2_ (99.9%) were purchased from Aladdin. Ca(NO_3_)_2_·4H_2_O (99%), Co(NO_3_)_2_·6H_2_O (99%), C_6_H_8_O_7_·H_2_O (99.5%), (CH_2_OH)_2_, HCl, HClO_4_, and ethanol were purchased from Shanghai Research Institute of Chemical Industry. Nafion (5%) was purchased from Sigma-Aldrich. All chemical reagents were used as received without further purification.

### Preparation of GO, HCPA and GO/HCPA Nanocomposite Papers

Ca_2_Co_x_Ir_1-x_O_4_ (x = 0, 0.1, 0.2, 0.4) was synthesized by a polymerized complex method. 3.12 g of Ca(NO_3_)_2_·4H_2_O and 2.8 g of C_6_H_8_O_7_·H_2_O were mixed with 50 mL of deionized water to form the solution A. Stoichiometric amounts of Co(NO_3_)_2_·6H_2_O and K_2_IrCl_6_ were dissolved in 40 mL of (CH_2_OH)_2_ to form the solution B: 0, 48, 96, and 192 mg of Co(NO_3_)_2_·6H_2_O mixed with 0.8, 0.72, 0.64, and 0.48 g of K_2_IrCl_6_ for x = 0, 0.1, 0.2, and 0.4. Then, the solution A was added dropwise in to the solution B under vigorous stirring. The resulting mixture was dried at 150 °C overnight to get solid powder as the precursor. This solid product was transferred to a crucible and heated in air at 200 °C (6 h), 300 °C (6 h), 500 °C (3 h) and 700 °C (6 h) with a heating rate of 1.7 °C min^−1^. Finally, the calcined solid powder was soaked in 1 M HCl for 6 h to remove oxides impurities. The remaining precipitate was washed several times with deionized water and ethanol, and dried in an oven to obtain CIO, 0.1Co–, 0.2Co–, and 0.4Co-CIO.

### Structural Characterizations and First-Principles Calculations

#### Structure Characterizations

Powder X-ray diffraction (XRD) patterns were collected on Philips X’ pert Pro Super diffractometer with Cu Kα radiation (λ = 1.5418 Å) with 2θ range of 10°–70°. The scanning electron microscopy (SEM) images were obtained on a JSM-6700 F scanning electron microscope operated at 5 kV. Transmission electron microscopy (TEM), high resolution transmission electron microscopy (HRTEM) and energy dispersive X-ray (EDX) spectra were performed on a JEOL-2100F microscope at an acceleration voltage of 200 kV. X-ray photoelectron spectroscopy (XPS) was acquired on an ESCALAB MKII instrument equipped with an Al Kα source (*hν* = 1486.6 eV), and all these spectra were calibrated with respect to C 1*s* at a binding energy of 284.5 eV. Magnetic properties were characterized by the vibrating sample magnetometer option of a Quantum Design physical property measurement system (PPMS) (Quantum Design, USA). The O *K*-edge and Co *L*-edge were performed at the Beamlines MCD-A and MCD-B (Soochow Beamline for Energy Materials) in National Synchrotron Radiation Laboratory (NSRL), which were measured in the total electron yield mode in a vacuum chamber (< 5 × 10^−8^ Pa).

#### Electrochemical Measurements

The electrochemical measurements were performed using a CHI 760E electrochemical workstation with a graphite rod and a saturated Ag/AgCl as the counter and reference electrodes, respectively. The working electrode was prepared by adding 5 μL of the catalyst ink (4 mg in 1 mL of 3:1 (volume ratio) deionized water and ethonal mix solvent) on the glassy carbon (GC; 3 mm in diameter) electrode. As comparison, commercial IrO_2_ catalyst was prepared by the same method. The sample loading was determined to be ~ 0.283 mg cm^−2^. The anodic linear sweep voltammetry (LSV) curves with *iR* drop compensation were measured in 1 M HClO_4_ at a rate of 10 mV s^−1^. All the potential values were calibrated to the reversible hydrogen electrode (RHE).

#### Evaluation of Electrochemically Active Surface Area (ECSA)

The ECSAs for as prepared catalysts were calculated based on:

1 $$ECSA = \frac{{C_{dl} }}{{C_{S} }}$$

where *C*_*S*_ is the specific capacitance and *C*_*dl*_ is the electrochemical double-layer capacitance. In this work, the reported value of *Cs* = 0.035 mF cm^−2^ was used to calculate the ESCA of our catalysts [31].

The *C*_*dl*_ was determined by measuring the non-Faradic capacitive current (in the window of 0.53–0.63 V) associated with double-layer charging from the scan rate (*v*: 2–10 mV s^−1^) dependence of CV. Finally, according to the equation of *i*_c_ = *vC*_*dl*_, *C*_*dl*_ equals to the slope of a plot of the double-layer charging current (*i*_c_) and the electrochemical double-layer capacitance.

#### Calculation of Turnover of Frequency (TOF)

TOF values were calculated according to the following equation:

2 $$TOF = \frac{{j \times A_{OX} }}{{4 \times e \times N_{A} }}$$

where *j* is the current density at an overpotential of 300 mV. *A*_*OX*_ is the total surface area of the catalyst deposited on the GC electrode. *e* is the electric charge carried by a single electron. *N*_*A*_ is the number of active sites. While calculating the *N*_*A*_, we assumed that all Ir atoms in the catalysts are active. Since only surface exposed Ir atoms participate into the OER process, the presented TOF values of different catalysts are underestimated.

#### Calculations of the Normalized Activity by ECSA and Ir Mass

ECSA-normalized current density for as prepared catalysts was calculated by:

3 $$ECSA - {\text{normalized current density}} = \frac{{\text{current density}}}{{{\mathrm{ECSA}}}}$$

The Mass activity (A g^−1^) values were calculated from the loading mass of iridium (*m)* and the corresponding current (*i*) at 1.5 V vs. RHE:

4 $$Mass\;activity = \frac{i}{m}$$

#### XAFS Measurements and Analysis

The XAFS (Co *K*-edge and Ir *L*_*3*_-edge) spectra were record at 1W1B beamline of Beijing Synchrotron Radiation Facility (BSRF, the storage rings were operated with a maximum electron current of 250 mA at 2.5 GeV), China. The energy of Co *K*-edge was calibrated by Co foil and the energy of Ir *L*_*3*_-edge was calibrated by Pt foil.

In-situ XAFS measurements were performed with Ir catalyst-coated carbon cloths in O_2_-saturated acidic solution by a smart homemade cell. To ensure full exposure of the Ir active sites to the electrolyte, the catalyst was uniformly and stably distributed over carbon cloth without obvious aggregation by continuous ultrasonication for 30 min in the electrolyte. The carbon cloth was taped with Kapton film on the back as the working electrode (~ 1 cm × 1 cm) to ensure all of the electrocatalyst reacted with HClO_4_ electrolyte. The collected XAFS data were processed using the ATHENA module implemented in the IFEFFIT software packages [31].

#### In-situ Raman Measurements

Raman spectroscopy (LabRam HR Evolution) at 633 nm excitation wavelength was used. A three-electrode epoxy pool cell with a 0.5 mm thick sapphire window was used for in-situ Raman measurements. To weaken the loss of Raman signal, the distance between the sapphire window of dry objective lens and the working electrode surface keeps a micrometer-scale gap. Acquisition time was set as 180 s for the spectral Raman shift ranging from 100 to 1300 cm^−1^ window. The Raman frequencies was normalized with the intensity of the characteristic peak of ClO_4_^−^ around 935 cm^‒1^.

#### Differential Electrochemical Mass Spectroscopy (DEMS) Measurements

DEMS measurements were carried out for 0.2Co-CIO and IrO_2_ using a QAS100 Plus device (Linglu Instruments, Shanghai). First, the catalysts were labelled with ^18^O isotope in 1 M HClO_4_ using H_2_^18^O as solvent at constant 1.6 V for 5 min. After that, ^18^O-labeled electrodes were rinsed with ^16^O water for five times to remove the remaining H_2_^18^O. Then, LSV cycles were performed in 1 M HClO_4_ using H_2_^16^O solvent in the potential range of 0.8–1.6 V versus RHE. at the same time, the gaseous products including ^36^O_2_, ^34^O_2_, and ^32^O_2_ were monitored by the mass spectrometer. The fraction of can be estimated from the integral areas of corresponding mass signals of these gaseous products.

#### XANES Calculation

The theoretical calculations on the Co *K*-edge XANES spectra were carried out with the FDMNES code in the framework of the real-space full multiple-scattering (FMS) scheme using a muffin-tin approximation for the potential [32–35]. The energy-dependent exchange–correlation potential was calculated in the real-space Hedin–Lundqvist scheme, and then the spectra were convolved using a Lorentzian function with an energy-dependent width to account for broadening due both to the core–hole width and the final state width. Especially for higher photoelectron energy, the plasmon collective interactions increase the Lorentzian width up to 7 eV. An input file with atomic positions of a supercell with a total of 63 atoms is prepared for 0.2Co-CIO. 20 at% Ir sites are randomly substituted by Co atom. A cluster of 6 Å radius was used for the self-consistency test and FMS, with satisfactory convergence being achieved. The energy scale was referenced to the Fermi level, whereas its zero pointed to the vacuum energy.

#### Details of First-principles Calculations

The spin-polarized density functional theory calculations were performed with Quantum Espresso software package [36]. The electron exchange–correlation was processed within the generalized gradient approximation (GGA) in the Perdew-Burke-Espresso (PBE) parametrization. The ion cores were described with the ultrasoft pseudopotentials. Hubbard-U correction was applied on Co and Ir atoms and the *U*_*eff*_ is 3.0 eV, which is determined by convergence tests. The *U*_*eff*_ were increased from a small value (e.g., 1.0 × 10^–3^ eV) until the calculated magnetic moments do not increase significantly. The van der Waals interaction correction was performed with the DFT-D3 method. The kinetic energy cutoffs of the plane wave and charge density were set to 75 and 500 Ry. The convergence tolerance of SCF was set to 1.0 × 10^–6^ eV atom^−1^. The positions of all the unfixed atoms were relaxed until the largest atomic force was less than 0.05 eV Å^−1^. The structure relaxations were performed with the BGFS method implemented in the Python library of Atomic Simulation Environment [37].

To more accurately describe the experimentally obtained OER performance, we adopted the surface-exposed active [IrO_6_] octahedra layer in slab models considering inevitable Ca leaching in acid and the well-maintained local structure properties of typical CIO and 0.2Co-CIO based O *K* edge and Co *L*_*2,3*_ edge XANES spectra. Firstly, a 7-layered slab model (Ca_36_Ir_27_O_93_ for pristine surface) with a vacuum layer of 15 Å along the z direction was adopted for the DFT calculations. The four bottom layers were fixed during the structure relaxation to simulate the bulk.

The adsorption energy of reaction intermediates is defined as:

5 $$E = E_{sub/M} - E_{sub} - E_{M}$$

where *E*_sub/M_, *E*_sub_ and *E*_M_ denote the total energies of an adsorbed system, a clean substrate, and an adsorbate at free state, respectively, each of which can be obtained from DFT calculations.

General steps of traditional adsorption evolution mechanism for OER:

$$\begin{gathered} OH^{-} + * \to *OH + e^{-} \hfill \\ OH^{-} + *OH \to *O + H_{2} O + e^{-} \hfill \\ OH^{-} + *O \to *OOH + e^{-} \hfill \\ OH^{-} + *OOH \to O_{2} + H_{2} O + e^{-} \hfill \\ \end{gathered}$$

General steps of the oxide path mechanism for OER:

$$\begin{gathered} OH^{-} + * \to *OH + e^{-} \hfill \\ OH^{-} + *OH \to *O + H_{2} O + e^{-} \hfill \\ 2*O \to O_{2} . \hfill \\ \end{gathered}$$

Gibbs free-energy of each step can be written as:

6 $$\Delta G = \Delta E + \Delta E_{ZPE} - T\Delta S + \Delta E_{U} - \Delta E_{pH}$$

where Δ*E* is the differential adsorbed groups energy. Δ*E*_ZPE_ and Δ*S* are respectively the difference between the adsorbed *H* and H_2_ molecules in zero-point energy and entropy. Especially, Δ*S* can be obtained by the approximate half of the entropy of H_2_ at standard conditions. *T* was chosen as 298 K.

7 $$\Delta E_{U} = - eU$$

in which *U* is the applied electron potential.

8 $$\Delta E_{pH} = kT\ln 10 \times pH$$

in which *k* is Boltzmann’s constant.

Thus, the differential energy of each adsorbed group is defined as:

9 $$\Delta E_{*OH} = \Delta E_{*OH} - E_{*} - \left( {\Delta E_{H20} - \frac{1}{2}E_{H2} } \right)$$

10 $$\Delta E_{*O} = E_{*O} - E_{*} - \left( {E_{H20} - E_{H2} } \right)$$

11 $$\Delta E_{*OOH} = E_{*OOH} - E_{*} - \left( {2E_{H20} - \frac{3}{2}E_{H2} } \right)$$

12 $$\Delta E_{*OO} = 2E_{*O} - E_{*}$$

in which $E_{*}$, $\Delta E_{*O}$, $\Delta E_{*OH}$, $\Delta E_{*OOH}$ and $\Delta E_{*OO}$ represent the energy of active site and active site binding with O, OH, OOH, OO respectively. The theoretical OER calculation was performed under pH = 0 based on the assumption proposed by J. K. Norskov et al. [38].

## Results and Discussion

### Morphology and Coordination Structure Characterizations

**Fig. 1:**
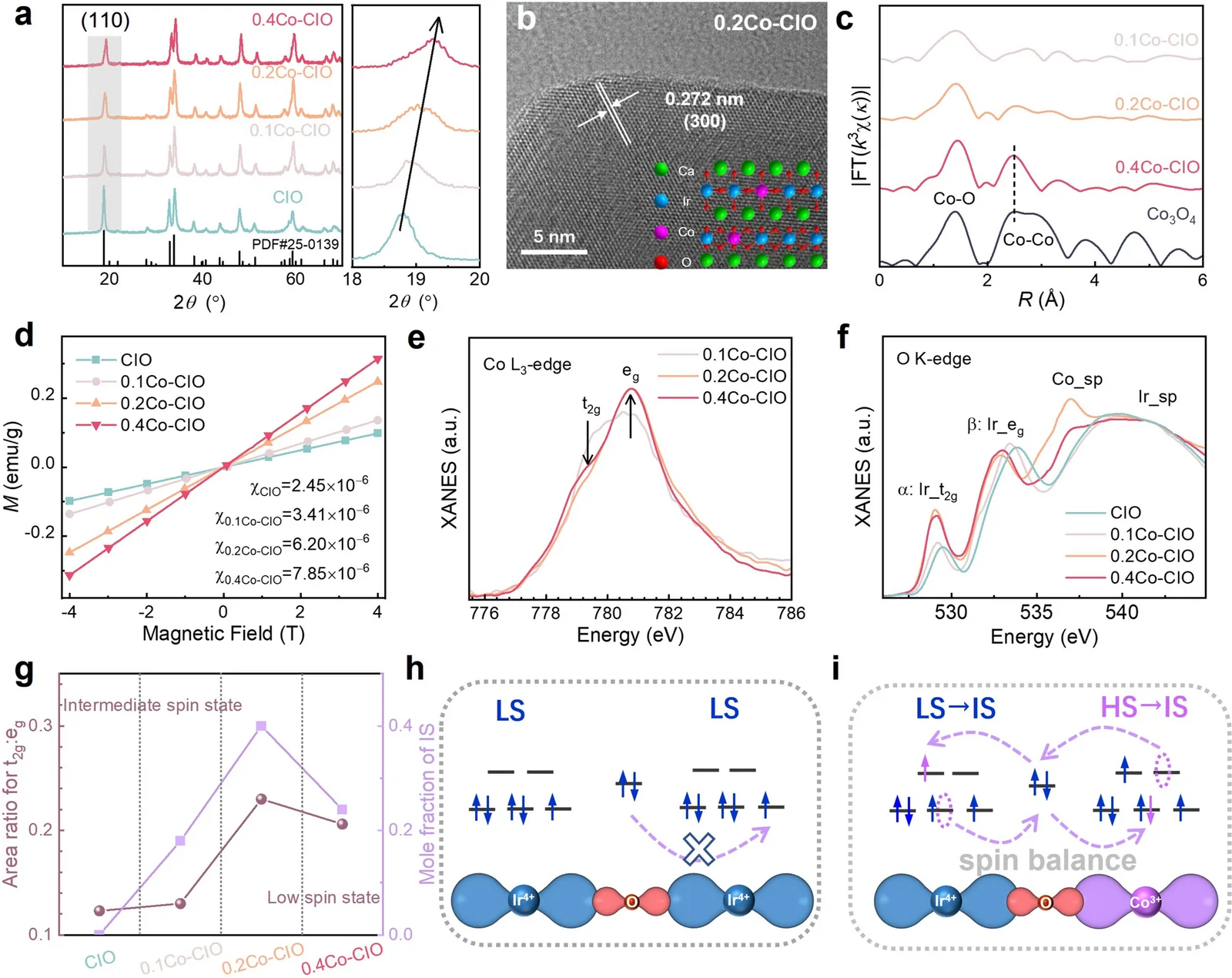
Structural characterization of pure CIO and Co-CIO catalyst.** a** XRD patterns for CIO, and 0.1Co-, 0.2Co-, 0.4Co- CIO samples. **b** HRTEM image of 0.2Co-CIO. Inset: Hexagonal 0.2Co-CIO crystal structure involving Co and Ir 0.2:0.8 long-range ordering. In this structure, both the Ir and Co atoms are octahedrally coordinated by six O atoms, and these octahedra are further edge connected with each other along the [001] direction to form 1D line with strong connection. **c** Co *K*-edge FT-EXAFS spectra of 0.1Co-, 0.2Co-, 0.4Co-CIO and Co_3_O_4_. **d** Hysteresis loops of the four samples recorded at 100 K. **e** Co *L*_3_-edge XAS spectra of 0.1Co-, 0.2Co-, and 0.4Co- CIO. **f** O *K*-edge spectra of the four samples. **g** The calculated ratio of *t*_2g_:*e*_g_ of as prepared samples with theoretical LS (0.125) and IS (0.330) state, and mole fraction of IS state Ir. **h** Illustration of the prohibitive spin flow between nearby LS Ir^4+^ ions due to the same spin electron distribution. **i** Illustration of spin balance between HS state Co and LS state Ir to give dual active sites of IS state Co and IS state Ir

The CIO sample displayed a Ruddlesden–Popper (214, RP) structure with $\mathrm{P}\overline{6 }2m$ hexagonal symmetry. Particularly, nearby IrO_6_ octahedron structure shared an edge to form a 1D Ir‒O‒Ir chain. The Co–CIO samples were synthesized via a polymerized complex method with different Co to Ir molar ratios of 0.1:0.9, 0.2:0.8, 0.4:0.6, followed by slow thermal decomposition in air. Scanning electron microscopy images of Co–CIO (Fig. S1) demonstrated similar average size of 40 nm in width and 20 nm in thickness, benefiting the utmost utilization of active metal sites. X-ray diffraction (XRD) patterns (Fig. 1a) of the obtained Co–CIO confirmed their high crystallinity and precluded the generation of secondary phases like Co oxides, wherein all diffraction peaks were attributed to the standard Ca_2_IrO_4_ phase. Additionally, the (110) peak gradually slight shift to higher angles are noticeable with increased Co amount (right panel of Fig. 1a), which confirmed that Co effectively enters the CIO lattice to substitute the Ir sites. As a result, the lattice constant decreased and provoked the scattering at higher angles. Typically, the energy dispersive X-ray spectroscopy (EDS) images (Fig. S2) of 0.2Co–CIO revealed that Ca, Ir, Co, and O were homogeneously distributed over the nanoparticles. And the high-resolution TEM image (Fig. 1b) demonstrated a typical inter-planar distance of 0.272 nm in 0.2Co–CIO, which corresponded to the (300) crystallographic plane of CIO, indicating a highly ordered atomic arrangement after Co incorporation. These results suggest the uniform substitution of Co dopants in the lattice of CIO sample (inset of Fig. 1b).

Specific local structure of Co–CIO was studied by extended X-ray absorption fine structure spectroscopy (EXAFS) characterizations at the Co K-edge and Ir *L*_*3*_-edge. According to the Fourier-transformed Ir L_3_-edge EXAFS (FT-EXAFS) spectra, Co–CIO and CIO presented similar shapes and trends in R space (Fig. S3), indicating that the well maintains of local [IrO_6_] octahedra structure after Co incorporation. Notably, the Co *K*-edge FT-EXAFS spectra of 0.1Co–CIO and 0.2Co–CIO exhibited dominant peaks at 1.4 Å, corresponding to the nearest shell coordination of the Co–O bond, which was similar to the nearest coordination environment of the Co_3_O_4_ reference (Fig. 1c). By comparison, 0.4Co–CIO exhibited another distinct peak at 2.5 Å in Co *K*-edge FT-EXAFS spectrum, which could be attributed to the Co‒Co bond due to excessive Co doping. It is noteworthy that combined with unchanged XRD results, Co‒Co bond stem from the Co–O–Co structure rather than the generation of second Co phase. Besides, the wavelet transform (WT) contour plots of 0.1Co-CIO and 0.2Co-CIO present only one intensity maximum at 4.2 Å^−1^ (Fig. S4), corresponding to Co − O coordination. There is another weak intensity maximum at 7.0 Å^−1^ for 0.4Co-CIO, which reconfirms a metallic Co‒Co coordination, similar like that of Co foil, CoO, and Co_3_O_4_ references. This result suggested that a moderate Co dopant concentration was the key to effectively regulating the delicate electronic structure of CIO.

Further, to better understand the precise location and distribution of Co dopants, Co *K* edge XANES simulation of optimal 0.2Co-CIO have been performed using as initio calculations via the FDMNES code. Firstly, an input file with atomic positions of a supercell with a total of 63 atoms is prepared. Specially, two Co atoms substitute the Ir sites to give a doped ratio of Co:Ir = 2:8 (inset of Fig. 1b). After the initial multiple scattering approach on a green potential, a convolution is performed to account for the core–hole broading and the spectral width of the final states. The simulated XANES (Fig. S5) matches the experimental one, although no relaxation of the input structure is performed. This fortifies the conclusion about the substitution of Co into the Ir sites and the formation of Janus Co − Ir interaction.

### Electronic and Spin Structure Characterizations

Further, we systematically investigated the influence of increased dopants concentration on the spin and electron interaction between Ir and Co atoms. The magnetic properties of Co–CIO and CIO were measured using a vibrating sample magnetometer. As shown in Fig. S6, the field-cooling magnetization curves were obtained under a certain external magnetic field (H = 1000 Oe). The magnetization of Co-CIO samples displayed a higher value than CIO, in which a more stable magnetic structure monotonically decreases with temperature. Moreover, the field dependence of the magnetization demonstrated similar linear profiles in the measured magnetic field range (Fig. 1d), suggesting the paramagnetic behavior of all samples at 300 K. The increased slope values with increased doping amount indicated the improved magnetic susceptibility of Co-CIO, which indicates the effective regulation of spin distribution by Co incorporation.

Subsequently, X-ray photoelectron spectroscopy (XPS) and XANES characterizations were performed to provide the change in electronic structure for Co-CIO and reveal the spin-state transition of Ir and Co atom. The oxidation state of Ir keep constant as approximately tetravalent based on the similar white line positions of Ir *L*_3_-edge XANES spectra for Co–CIO, CIO, and IrO_2_ (Fig. S7). While the Co K-edge absorption energy position of all Co–CIO samples was similar to that of standard Co_2_O_3_ (Fig. S8), suggesting that the average valence state was Co^3+^. Moreover, Ir 4*f* XPS spectra (Fig. S9a) of Co–CIO did not exhibit obvious variations in these doublet peaks as compared to CIO, indicating a consistent valence state of bulk and surface Ir^4+^. And Co 2*p* XPS spectra (Fig. S9b) exhibited one set of doublets at 796.9 and 780.6 eV, which is attributed to Co^3+^. These results suggested that Co^3+^ dopants are uniformly distributed to effectively regulate the spin state of nearby Ir atoms with constant + 4 valence state. Based on the charge neutrality, lattice oxygen loss was expected in the Co–CIO structure (Fig. S10), wherein Co atoms were most likely under-coordinated.

XANES characterizations at the Co *L*_2,3_-edge and O *K*-edge, which are highly sensitive to the electronic/spin structure of surface active sites, were performed to further confirm the spin-state transitions. The Co *L*_2,3_-edge spectra (Fig. S11) exhibited two characteristic peaks at approximately 780 and 795 eV (denoted as *L*_3_ and *L*_2_, respectively), which correspond to the transitions from 2*p*_*1/2*_ and 2*p*_*3/2*_ to 3*d*, respectively [35, 39]. Compared to Co^3+^ compound references [40, 41], the dominant main peak (*L*_3_) at approximately 780.7 eV in the Co *L*_2,3_-edge spectrum for Co–CIO indicated the trivalent state of surface Co atoms, which was consistent with those in the bulk. More importantly, the shoulder and main peaks at approximately 779 and 780.7 eV, respectively, corresponded to electron transitions from the *p* orbital to *t*_2g_ and *e*_g_ orbitals, which directly conveyed the unoccupied states of the *t*_2g_ and *e*_g_ orbitals and indicated their spin-state transitions (Fig. 1e). In the HS state, 3*d t*_2g_ and *e*_g_ orbital filling of Co^3+^ ions generated a *t*_2g_^4^*e*_g_^2^ configuration. However, the *t*_2g_ orbitals were increasingly occupied while the *e*_g_ orbitals were increasingly less occupied as the IS state of Co^3+^ ions (*t*_2g_^5^*e*_g_^1^) emerged, resulting in decreased *t*_2g_ and increased *e*_g_ peak intensities in Co *L*_3_-edge XANES spectra. The Co *L*_2,3_-edge spectrum for 0.1Co–CIO (Fig. S11) was similar to that for the HS Co^3+^ reference, demonstrating that the Co^3+^ dopants in 0.1Co–CIO were mainly in the HS state. With the increase of Co amount, the normalized intensity of the shoulder peak was reduced in the order of 0.1Co–CIO > 0.4Co–CIO > 0.2Co–CIO (Fig. 1e), demonstrating the dominant IS state of Co^3+^ in 0.2Co–CIO. The relatively lower amount of IS Co^3+^ in 0.4Co–CIO than 0.2Co–CIO may have been associated with the formation of a local Co‒O‒Co structure, as manifested by Co K edge FT-EXAFS combined with WT analysis in Figs. 1c and S4.

Figure 1f presents similar O *K*-edge spectra for Co–CIO and CIO, with two characteristic peaks located at 529.5 and 533.9 eV (denoted as *α* and *β*, respectively), which were assigned to hybridization of the O 2*p* state to Ir 5*d t*_2g_ and *e*_g_ states due to splitting of the octahedral field. With the substitution of Co^3+^ at the Ir^4+^ site, both peaks shifted to lower energy positions owing to the formation of Ir‒O‒Co bonds [15, 42]. The spin state of Ir^4+^ could be determined from the statistical ratio of the integrated intensities of the two peaks, namely, the I_t2g_/I_eg_ ratio [43, 44]. Detailed analysis method can be found in the description of Fig. S12. In the case of Ir^4+^ in the LS state (*t*_2g_^5^*e*_g_^0^), a theoretical I_t2g_/I_eg_ ratio of 0.125 was expected, which was consistent with the calculated I_t2g_/I_eg_ ratio of 0.126 for IrO_2_ and 0.123 for CIO (Figs. S13, S14a and Table S1), validating the expected estimation of Ir spin state through I_t2g_/I_eg_ ratio method. For Co–CIO, the I_t2g_/I_eg_ ratio gradually increased with Co dopant incorporation (Fig. S14b-d and Table S1), suggesting that the spin state of partial Ir ions transformed from the LS to IS states (t_2g_^4^e_g_^1^). Using the calculated I_t2g_/I_eg_ ratio, the spin states of Ir^4+^ ions were estimated to be 2 vol% IS + 98 vol% LS, 50 vol% IS + 50 vol% LS, and 40 vol% IS + 60 vol% LS for 0.1Co–CIO, 0.2-Co–CIO, and 0.4Co–CIO, respectively (Table S1). Employing the chemical formula of each sample, the estimated mole fractions of Ir^4+^ atoms in the IS state (Fig. 1g) were approximately 0.18, 0.40, and 0.24 for 0.1Co–CIO, 0.2-Co–CIO, and 0.4Co–CIO, respectively. These results suggested the effective spin regulation of two adjacent Ir atoms centered around the Co dopant in the 1D Ir‒O‒Co chain.

Figure 1h, i illustrated the spin balance effect after Co doping for the regulated OER performance. For CIO with LS Ir state (Fig. 1h), identical spin distribution of nearby Ir atoms forbidden the electron flow process. Under the application of overpotentials during OER process, electron hopping consumes energy from Ir^4+^–O–Ir^4+^ to Ir^4+^–O–Ir^5+^ with higher oxidation states. Then, OER current flow along the 1D chain of edge-sharing [IrO_6_] octahedra. While when HS Co was doped to form a Janus Ir (LS)–Co (HS) structure, electron hopping from the half-filled Co *e*_g_ orbitals to fully empty Ir *e*_g_ orbitals occurred thermodynamically, accompanied by electron transfer from nearly filled Ir *t*_2g_ orbitals to partially empty Co *t*_2g_ orbitals. Therefore, the Janus Ir–Co magnetic–coupling structure realized spin balance between LS Ir (t_2g_^5^e_g_^0^) and HS Co (t_2g_^4^e_g_^2^), contributing to the optimal IS state of Ir and Co atoms. Metal centers with *e*_g_^1^ orbital filling were easily accessible for the adsorption/desorption of oxygen-containing intermediates via *e*_g_–*p* orbital coupling, and reduced *t*_2g_ orbital filling in the Ir‒O‒Co bonds would accelerate electron transfer to promote OER performance. It is noteworthy that edge-sharing octahedra play a key role for spin balance when compared with face- or corner-sharing octahedra, which make the electron interaction too strong or too weak, respectively. Besides, we prefer Co rather than Fe or Ni as the dopant considering that Ni usually present LS Ni^3+^ in octahedron, which is unable to trigger spin balance, while activated IS Fe^3+^ shares the same t_2g_ occupancy with nearby IS Ir^4+^ (t_2g_^4^e_g_^1^), which impede charge transfers and make bad OER efficiency.

### Evaluation of OER Performances

**Fig. 2:**
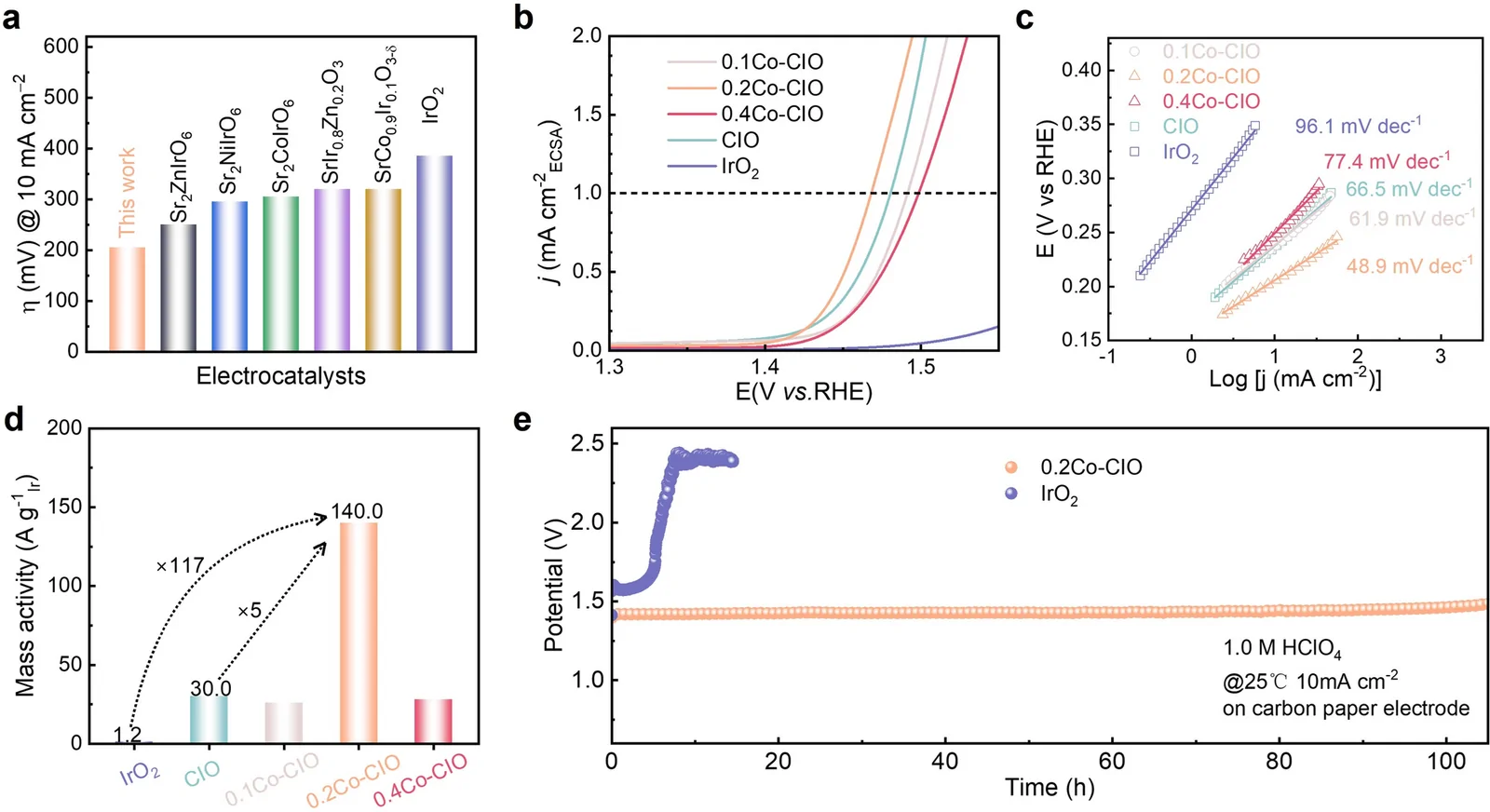
Electrochemical OER properties of Co-CIO, CIO and IrO_2_ in 1.0 M HClO_4_. **a** Comparison of the overpotentials at 10 mA cm^‒2^ with selected Ir based perovskites systems. **b** ECSA normalized LSV curves. **c** Tafel slope. **d** Comparison of the mass activity under 1.45 V versus RHE. **e** Chronopotentiometry curves of 0.2Co-CIO and IrO_2_ at 10 mA cm^‒2^ (with IR compensations). Carbon paper was used as the catalyst support for stability test

Subsequently, the electrocatalytic properties of Co–CIO, CIO, and IrO_2_ for OER were evaluated in 1 M HClO_4_. Linear sweep voltammetry (Fig. S15) revealed that 0.2Co–CIO needed a very small overpotential of 205 mV to reach a current density of 10 mA cm^‒2^, which was superior to most reported Ir-based perovskites and recently reported Ir-based oxides, including Sr_2_CoIrO_6_ [45, 46], Sr_2_NiIrO_6_ [46], and SrZn_0.2_Ir_0.8_O_3_ [47], as well as IrCo_ae [48] and 5Ir-Co_3_O_4_-bilayer [49] etc. (Fig. 2a and Table S3). With 20 at% previous Ir substituted by Co to reduce catalyst cost, this drastically reduced overpotentials of nearly 30 mV for 0.2Co-CIO compared to CIO demonstrate the particularly positive regulation of Co dopants to Ir oxides. To exclude the influence of geometric effects, the current densities of as-prepared samples were normalized to the electrochemically active surface area (*j*_ECSA_) via cyclic voltammetry (Figs. S16 and S17). As shown in Fig. 2b, 0.2Co–CIO exhibited the best catalytic activity among the as-prepared samples. Moreover, the outstanding OER activity of 0.2Co–CIO was reflected by the smallest Tafel slope (48.9 mV dec^‒1^) compared to 0.1Co–CIO (61.9 mV dec^‒1^), 0.4Co–CIO (77.4 mV dec^‒1^), CIO (66.5 mV dec^‒1^), and IrO_2_ (96.1 mV dec^‒1^), suggesting its faster OER kinetics (Fig. 2c). Electrochemical impedance spectroscopy (Figs. S18 and S19) revealed the accelerated charge transfer kinetics of Co–CIO after Co incorporation. Notably, 0.2Co–CIO exhibited the lowest charge transfer resistance (R_ct_) value among the as-prepared samples (Table S3), demonstrating that the local electronic structure was optimized through the Ir‒O‒Co configuration.

Further, the mass activity (normalized by Ir mass) of 0.2Co–CIO was calculated to be 140 A g^‒1^ under 1.45 V versus RHE (Fig. 2d), which is 117 times that of commercial IrO_2_ (1.2 A g^‒1^) and 5 times that of CIO (30.0 A g^‒1^), indicating the more active Ir sites after the construction of local Co − O − Ir structure. The Ir mass activity of 0.2Co–CIO under 1.53 V with that of recently developed Ir-based catalysts is presented in Table S3, demonstrating that 0.2Co–CIO delivered the highest Ir mass activity of 1110 A g_Ir_^‒1^, which was almost 140 times higher than that of IrO_2_ (8 A g_Ir_^‒1^). Moreover, the TOF was calculated to be 1.53 V. The Ir site in 0.2Co–CIO exhibited a high TOF of 2050 h^‒1^, which was approximately 3 orders of magnitude higher than that in CIO (720 h^‒1^) and 200 times higher than that in IrO_2_ (11 h^‒1^). The electrochemical stability of 0.2Co–CIO was assessed via chronopotentiometry. Contrary to IrO_2_, 0.2Co–CIO exhibited no obvious decrease in activity during continuous operation for 100 h (Fig. 2e), demonstrating its robustness in long-term OER operation. Moreover, 0.2Co–CIO operated stably for approximately 36 h under harsh acidic conditions, even at a constant current density of 50 mA cm^‒2^, indicating its excellent acidic OER stability (Fig. S20).

The dissolution of metal ion from 0.2Co-CIO and CIO during OER process is assessed by inductively coupled plasma optical emission spectroscopy (Fig. S21). At the early stage (0–100 cycles), fast Ca and Ir dissolution is observed from CIO. The dissolution of Ca likely induces such high dissolution rate of Ir. In the following cycles, the dissolution rate of Ir from CIO is found slowed down, which is due to the formed Ir-rich surface layer. After Co doping, the speed of Ca and Ir dissolution from 0.2Co-CIO apparently slowed down, which suggests the hinder effect of strong interaction between Co and Ir atom. Although metal leaching and surface reconstruction is inevitable for cycled 0.2Co-CIO, EDS line-scan spectra show the coexistence of Co and Ir in the near-surface region similar as pristine one (Fig. S22). HRTEM image also indicates the well-maintained lattice fringes with minor crystalline particles on the surface after 2000 CV cycles (Fig. S23). Furthermore, XANES spectra at O *K*-edge and Co *L*_*3*_ edge present negligible variations for optimal 0.2Co-CIO during certain CV cycles test (Fig. S24). Detailed fitting result of the pre-edge peaks in O *K* edge XANES spectra indicated the slightly reduced ratio of IS Ir amount from pristine 0.4 to 0.35 mol after 2000 CV cycles test (Fig. S25 and Table S1), which is due to the inevitable dissolution of surface Co atoms in acid. These results confirmed the relatively stability of Janus Co − Ir structure on surface for the long durability under 10 mA cm^‒2^ of 0.2Co-CIO for 100 h in acid.

### Mechanism Insights into OER Process

**Fig. 3:**
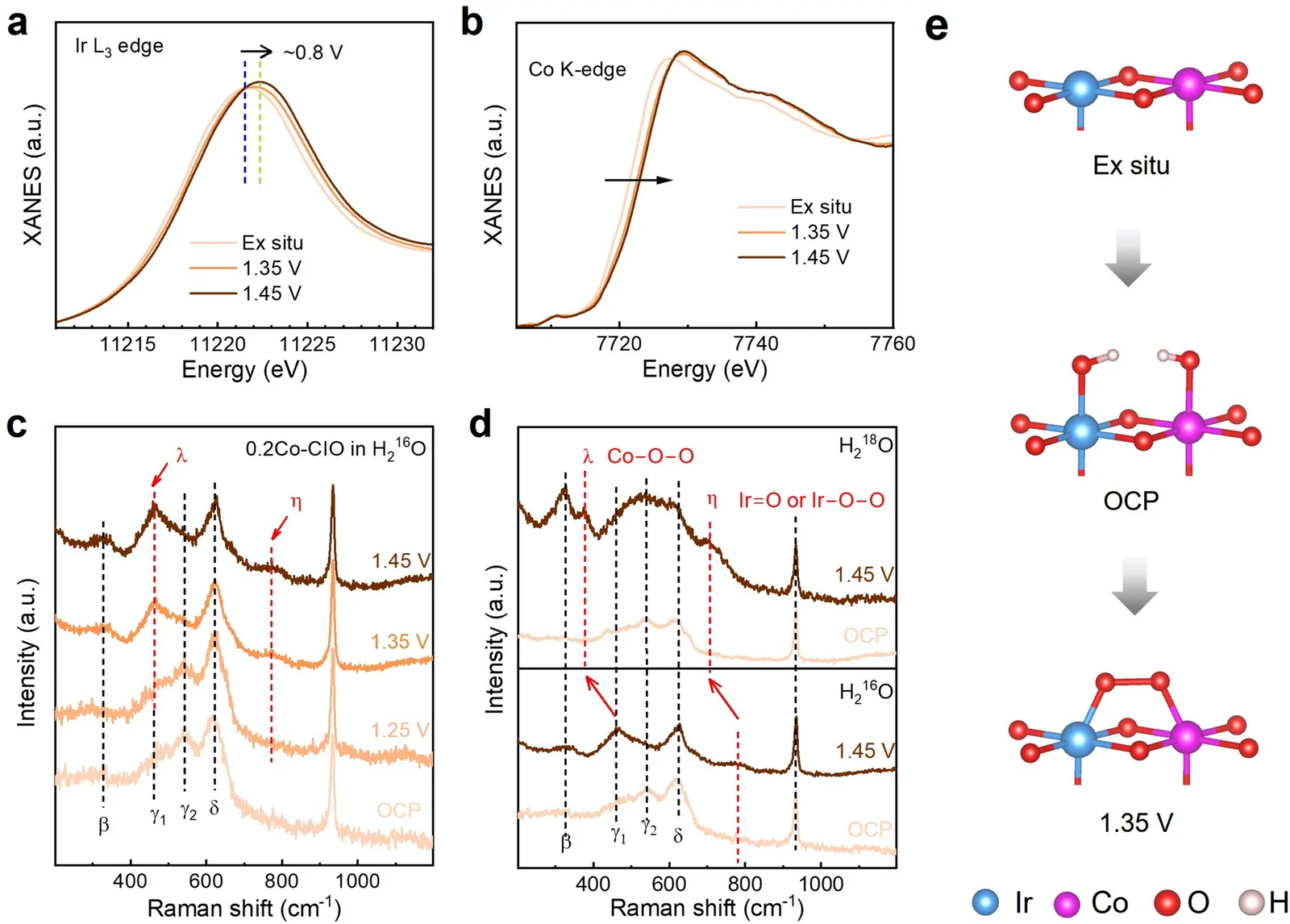
In-situ spectroscopies studies of 0.2Co-CIO. In-situ XANES spectra of **a** Ir *L*_*3*_ edge and **b** Co *K*-edge on 0.2Co-CIO. **c** In-situ Raman spectra of 0.2Co-CIO in H_2_^16^O. **d** Comparison of in-situ Raman spectra of 0.2Co-CIO in H_2_^16^O (bottom panel) and H_2_^18^O (top panel). **e** The atom model of adsorbed *OO formation on hetero-dual-atom Ir-Co active sites

To learn more about the active sites of 0.2Co–CIO and examine its chemical state evolution during the OER process, in situ XANES characterizations at the Ir *L*_3_-edge and Co *K*-edge were performed. As shown in Fig. 3a, the white line positions at the Ir *L*_3_-edge for 0.2Co–CIO shifted to more positive values under the applied potentials relative to the ex situ state, suggesting that Ir atoms were oxidized and possessed an increased valence state during OER. The white line position at the Ir *L*_3_-edge for 0.2Co–CIO shifted to a higher energy by 0.8 eV under an applied voltage of 1.45 V, which was 50 mV lower than that for CIO (Fig. S26), confirming the easier electron transition of Ir atoms in 0.2Co–CIO after Co incorporation. The average valence state of Ir atoms was estimated to be 4.6 under 1.45 V according to the white line position versus valence state standard curve (Fig. S27). This finding suggested that the Ir sites of 0.2Co–CIO were efficiently activated to achieve enhanced OER performance. Meanwhile, the absorption edge at the Co *K*-edge XANES shifted to a higher energy by 1.7 eV during the OER process (Fig. 3b), indicating that the Co valence state increased to approximately 3.7 compared to standard references (Fig. S28). The increased oxidation states of Ir and Co atoms suggested the presence of synergetic hetero-dual active centers in the spin-regulated Ir‒O‒Co units in 0.2Co–CIO [28]. It is noteworthy during OER process, dual active sites of Co and Ir transfer electrons via the metal t_2g_-O 2*p* orbital, while e_g_ occupancy keeps stable to facilitate the adsorption–desorption of intermediates. Therefore, Co dopant regulates both t_2g_ and e_g_ orbital occupancy, which co-promote the OER activity.

^18^O-labeling experiments combined with in situ Raman spectroscopy were employed to identify the oxygen-containing species during the OER process, thereby better understanding the active interfacial structure evolution and providing deeper insight into the OER reaction mechanism. Figures S29 and 3c present the Raman spectra for CIO and 0.2Co–CIO immersed in 1.0 M HClO_4_ at selected applied potentials. The peak at 933 cm^‒1^ was attributed to ClO_4_^‒^, while those at 327, 460, 542, and 621 cm^‒1^ were attributed to Ir–O vibrations [11]. When the applied potential was increased, an additional peak (*η*) at approximately 770 cm^‒1^ began to appear at 1.5 and 1.35 V for CIO and 0.2Co–CIO, respectively (Fig. 3c, d). Moreover, the intensity of the *η* peak increased with increasing sweeping potential, confirming the higher OER activity of 0.2Co–CIO compared to CIO. Considering that = O species appears at higher wavenumber (800–900 cm^–1^) than O–O [50] and is usually accompanied with super-high metal value like Ir^6+^, the *η* peak at 770 cm^‒1^ was uniquely attributed to Ir‒OO.

Notably, the Raman spectrum for 0.2Co–CIO displayed a distant peak (*λ*) at approximately 455 cm^‒1^, the intensity of which increased with increasing sweeping potential (Fig. 3c). The *λ* peak was not observed for CIO during OER, indicating that it was associated with the Co site in 0.2Co–CIO. Furthermore, ^18^O isotopic substitution was performed for 0.2Co–CIO (Fig. S30). Accordingly, the *λ* peak presented a shift of approximately 64 cm^‒1^ (Fig. 3d), indicating that the Co^18^O^18^O species was formed via isotope exchange of two ^16^O atoms. Similar spectral evidence for the O‒O stretch of CoOO has been clearly identified in the literature [51–53]. Therefore, spin balance over the Janus Ir–Co magnetic structure resulted in direct O‒O coupling in 0.2Co–CIO via the SOPM route (Fig. 3e), which generally yields faster water oxidation kinetics than the traditional AEM route.

We then conducted in situ differential electrochemical mass spectroscopy (DEMS) measurements using the isotope ^18^O to investigate the direct coupling of O − O on 0.2Co-CIO compared to commercial IrO_2_. We labeled the catalyst surface with ^18^O and measured the evolved O_2_ in H_2_^16^O solvent. The ratio of ^36^O_2_ generated on IrO_2_ come from the natural abundance. While for 0.2Co-CIO, the ratio of ^36^O_2_ is much higher than that on IrO_2_ (Fig. S31), reflecting the combination of ^18^O from nearby active sites. Therefore, the direct O − O coupling give rise to the peak at 455 cm^‒1^ in Raman spectra.

### DFT Calculation

**Fig. 4:**
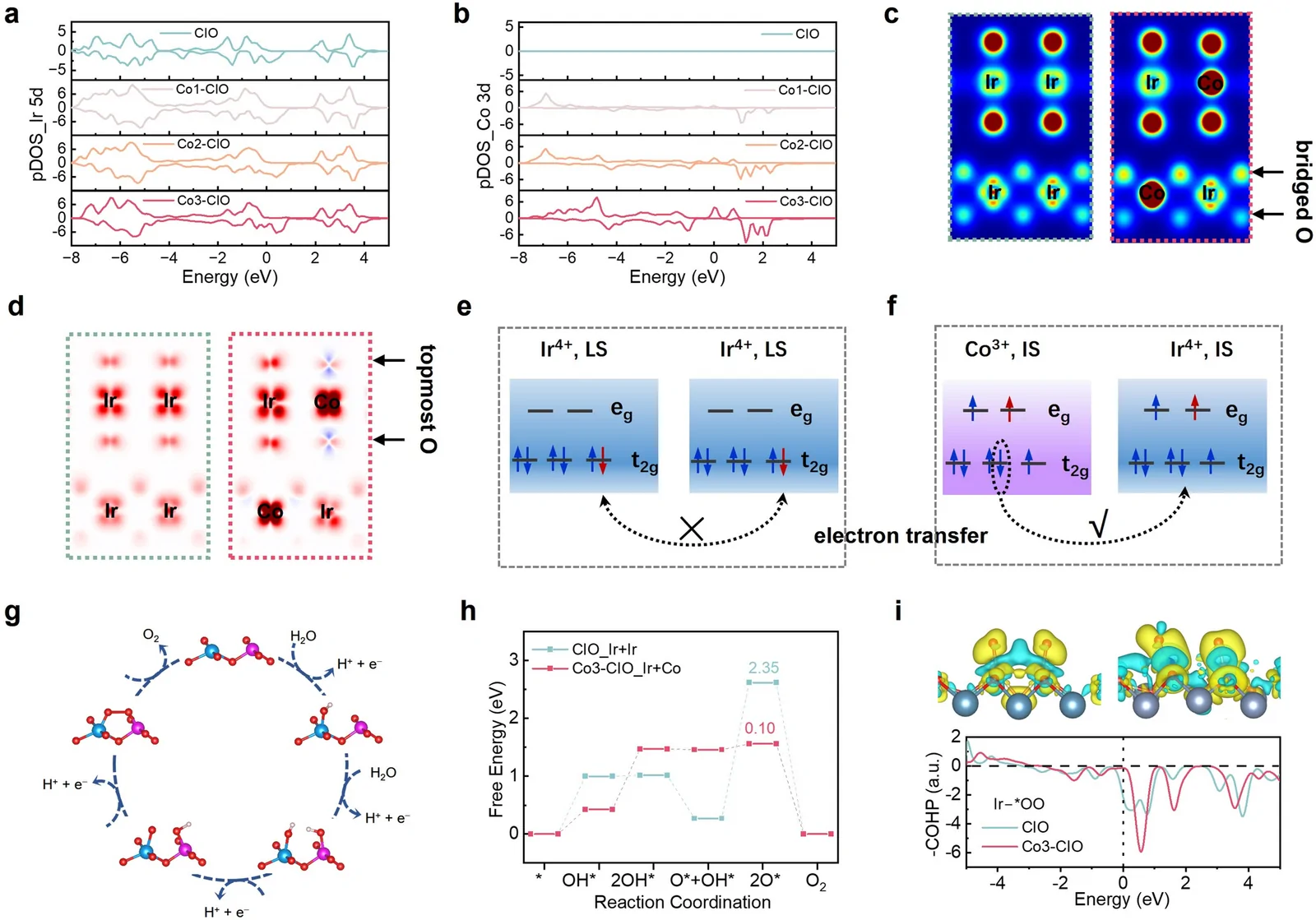
Theoretical investigation of the OER activity in CIO with the spin regulation by Co incorporation. The spin-resolved PDOS of **a** Ir atom and **b** Co atoms in CIO with various Co doping. **c** Electron density and **d** spin density of CIO (left) and Co3-CIO (right). Schematic representation of the electron occupancy and electron transfer of **e** CIO and **f** Co3-CIO. **g** OER process through SOPM pathway. **h** Free energy profile at 1.23 V for the OER pathway with ΔE = 0.93 eV on the Ir-Ir sites of CIO and with ΔE = 0.73 eV on the Co-Ir sites of Co3-CIO. **i** Top panel: the charge density difference of adsorbed *OO onto Ir‒Ir (left) and Co‒Ir (right) site. The bottom panel: the COHP of adsorbed *OO onto Ir‒Ir and Co‒Ir sites

To better understand the spin-state regulation of Ir/Co active sites and changes in electrocatalytic activity, first-principles theoretical calculations were performed on slab models with different Co^3+^ amounts, denoted as CIO, Co1–CIO, Co2–CIO, and Co3–CIO (Fig. S32). More detailed discussion about the selected structure models with various Co concentration can be found in supporting information. The projected densities of states (PDOS) of Ir 5*d* and Co 3*d* are presented in Fig. 4a, b. For CIO, the PDOS of Ir 5*d* was almost symmetrical due to the LS state of Ir^4+^. After Co doping, the higher energy shift of the spin-down domain indicated that this symmetry was gradually broken with increased Co^3+^ amount, resulting in the increased spin state of Ir^4+^. This result aligned with the enhanced magnetic susceptibility of Co–CIO compared to that of CIO (Fig. 1d). Meanwhile, the spin-up and spin-down domains of the PDOS of Co 3*d* were asymmetrical for Co1–CIO (Fig. 4b), suggesting the HS state of the Co^3+^ dopants. The higher energy shift of the spin-up domain indicated that the asymmetry of the PDOS of Co 3*d* gradually decreased with increased Co^3+^ amount, suggesting the decreased spin state of Co^3+^. Subsequently, we calculated the magnetic moment changes, finding that the average magnetic moments of Ir atoms gradually increased from 0.403 to 0.443, 0.479, and 0.494 *μ*B for CIO, Co1–CIO, Co2–CIO, and Co3–CIO, respectively. Meanwhile, the average magnetic moments of Co atoms gradually decreased from 2.715 to 2.120 and 1.479 *μ*B for Co1–CIO, Co2–CIO, and Co3–CIO, respectively. Therefore, the spin-state changes could be ascribed to spin balance over the Janus Ir–Co structure in Co–CIO.

Furthermore, we calculated the electron and spin densities of the metal centers. The electronic density of Ir was almost unchanged after Co doping (Figs. 4c and S33), which was identified as the unchanged valence state of Ir^4+^ via XPS and Ir *L*_*3*_-edge XANES characterization. Meanwhile, the bridged oxygen (O_bri_) atoms presented an asymmetric electron distribution, demonstrating spin–orbit interaction via the Co‒O_bri_‒Ir bonds in Co–CIO. While Ir and Co atoms exhibited gradually increased and decreased spin densities (Figs. 4d and S34), respectively. The spin regulation of both t_2g_ and e_g_ orbital results in the enhanced OER performance, as compared in Fig. 4e, f. For CIO with LS Ir, the external electron from intermediates will occupy the last vacancy t_2g_ orbital of LS Ir (Fig. 4e). However, the identical distribution of spin electron in t_2g_ orbital make the internal charge transfer difficult along the 1D Ir − O − Ir chain for CIO. While for Co3-CIO (Fig. 4f), the external electron from intermediates will occupy the vacancy e_g_ orbital in a higher energy level [15, 54]. Simultaneously, the reduced electron occupancy in the t_2g_ orbitals of IS Ir induced electron delocalization, which was beneficial to the internal charge transfer process. This spin reconfiguration also led to a structural transition, as shown in Fig. S35. The structural model of Co–CIO with increased Co doping after optimization exhibited a gradually decreasing M‒M distance from 3.254 Å for the Ir‒Ir bond in CIO to 3.128, 3.115, and 3.068 Å for the Ir‒Co bond in Co1–CIO, Co2–CIO, and Co3–CIO, respectively. This change facilitated O–O coupling over the spin-regulated Ir‒O‒Co units, which acted as dual active sites for synergistic catalysis with faster OER kinetics.

To verify the spin balance effect over the entire OER process, the Gibbs free energies of each step through the possible AEM and SOPM routes for CIO and Co3–CIO were compared under the applied potential of 1.23 V. Figure 4g presents the related OER steps and reaction intermediates in the SOPM route. As shown in Figs. 4h and S36, the energetic barriers of each RDS are denoted in the diagrams, which were utilized to estimate the reaction pathway. The calculations on single Ir sites indicated that the RDS for CIO was primarily derived from the adsorption of *OH as the first step for AEM, with an energy barrier of 1.00 eV. Moreover, direct O‒O coupling over two adjacent Ir active sites through SOPM had a slightly lower energy barrier of 0.93 eV. By contrast, the spin-regulated Ir‒O_bri_‒Co centers in Co3–CIO favored the SOPM route, wherein the *OO intermediate simultaneously bridged with Ir and Co sites, with the lowest energy barrier of 0.73 eV compared to that for *OOH on single Ir (1.65 eV) and for *OH on single Co (1.18 eV). The results indicated that the catalytic activity of Co–CIO was related to the changed OER pathway due to spin balance over the Janus Ir–Co magnetic structure. The results indicated that the catalytic activity of Co–CIO was related to the changed OER pathway due to spin balance over the Janus Ir–Co magnetic structure.

The charge density difference of adsorbed *OO on LS and IS active sites is calculated in the top panel of Fig. 4f to help better understand the effect of spin regulation on free energy. The yellow and blue isosurfaces correspond to the electron-depletion and electron-increase zones, respectively. When *OO was attracted to the catalytic surface, the unpaired O *2p* orbitals were likely to hybridize with IS Ir *5d* and Co *3d* orbitals. Compared with two adjacent LS Ir active sites in CIO, the blue zone for 3Co–CIO extended to the active IS Ir‒O_bri_‒Co centers, increasing the electron occupancy of the *e*_*g*_ orbital of Ir to 3.84 (IS) from 3.79 (LS). The bonding interaction between active sites and adsorbed *OO exhibited a mixed ionic-covalent characteristic owing to the energetic similarity (covalency) and spatial overlap between Ir *5d* and O *2p* orbitals, which played a primary role in determining the reactive activity. To confirm this perspective, the crystal orbital Hamilton population (COHP) was calculated to compare the bonding character before and after Co doping. Differing from the Ir–*OO–Ir bonding contribution in the LS state, a stronger antibonding state appeared at the Fermi level, confirming that electrons from the Ir 5*d* orbital partially transferred to the unfilled O 2*p* orbital easily. Moreover, the stability of the Ir–OO bond in the IS state was lower than that in the HS state, leading to a lower reaction activation energy.

## Conclusions

Herein, we demonstrate that spin-state regulation of LS Ir^4+^ sites can be effectively realized via a spin balance strategy through a Janus Ir–Co structure with HS Co^3+^ substitution doping in Ir-based oxides. Charge transfer in the spin-regulated Ir‒O_bri_‒Co units and orbital interactions with intermediates display spin-dependent reaction kinetics, with the optimal 0.2Co–CIO demonstrating a low overpotential of 205 mV and maintaining high activity during OER operation for 100 h. Moreover, the ultrahigh mass activities and TOF of 0.2Co–CIO are approximately 1110 A g^‒1^ and 2050 h^‒1^ at 1.53 V versus RHE, respectively, which is 140–190 times higher than that of commercial IrO_2_. In situ experiments combined with DFT calculations reveal that Co–CIO prefers an effective SOPM to achieve superb OER activity in acidic media, which differs from CIO. Our results provide new insight to further improve the understanding of the internal relationship within spin-related electrocatalysts.

## Supplementary Information

Below is the link to the electronic supplementary material.

Supplementary file1 (DOCX 23128 KB)
