# Supplementary material for: Spin Balance Over Janus Ir-Co Magnetic Atoms for Efficient Acidic Water Oxidation
Source: Nanomicro Lett. 2026 Jan 28;18:227. doi: 10.1007/s40820-026-02082-2 (PMC12852515; doi:10.1007/s40820-026-02082-2)
Supplement: Supplementary file 1 — Supplementary file1 (DOCX 23128 KB) [file 40820_2026_2082_MOESM1_ESM.docx]

Supporting Information for

**Spin Balance over Janus Ir-Co Magnetic Atoms for Efficient Acidic Water Oxidation**

Na Li^1^, Weiren Cheng^2,^ *, Yuying Liu^1^, Ruiqi Liu^1^, Sihua Feng^1^, Huijuan Wang^3^, Liyang Lv^1^, Chenglong Liu^1^, Jin Ma^1^, Chao Wang^1,^ *, Wensheng Yan^1,^ *

^1^ National Synchrotron Radiation Laboratory, University of Science and Technology of China, Hefei, Anhui 230029, People’s Republic of China

^2^ Key Laboratory of Precision and Intelligent Chemistry, Department of Materials Science and Engineering, University of Science and Technology of China, Hefei, Anhui 230026, People’s Republic of China

^3^ Material Test and Analysis Lab, Energy and Materials Science Experiment Center, University of Science and Technology of China, Hefei, Anhui 230029, People’s Republic of China

*Corresponding authors. E-mail: : [weiren@ustc.edu.cn](mailto:weiren@ustc.edu.cn) (Weiren Cheng); [chaowng@ustc.edu.cn](mailto:chaowng@ustc.edu.cn) (Chao Wang); [ywsh2000@ustc.edu.cn](mailto:ywsh2000@ustc.edu.cn) (Wensheng Yan)

**Supplementary Figures and Tables**

**Fig. S1** SEM images and particle distribution for **a** CIO, **b** 0.1Co-, **c** 0.2Co- and **d** 0.4Co-CIO

**Fig. S2** EDX maps of 0.2Co-CIO. Ca, iridium, oxygen, and cobalt are displayed in green, light blue, red, and magenta, respectively


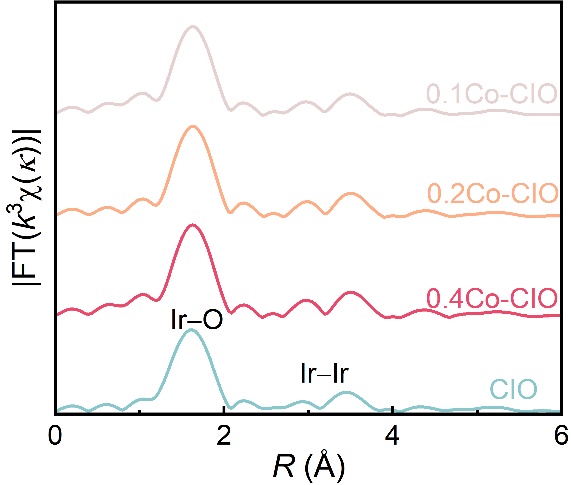


**Fig. S3** Ir *L*_3_-edge FT-EXAFS spectra of 0.1Co-, 0.2Co-, 0.4Co-CIO and CIO

**Fig. S4** WT for the *k*^2^-weighted EXAFS signal

**Fig. S5** Comparison between the simulated and the experimental absorption spectrum for 0.2Co-CIrO with a random distribution. The dotted vertical lines indicate the positions of three main absorption peaks with a weak pre-edge peak. The illustration of constructed supercell model can be found in the inset of Fig. 1b

**
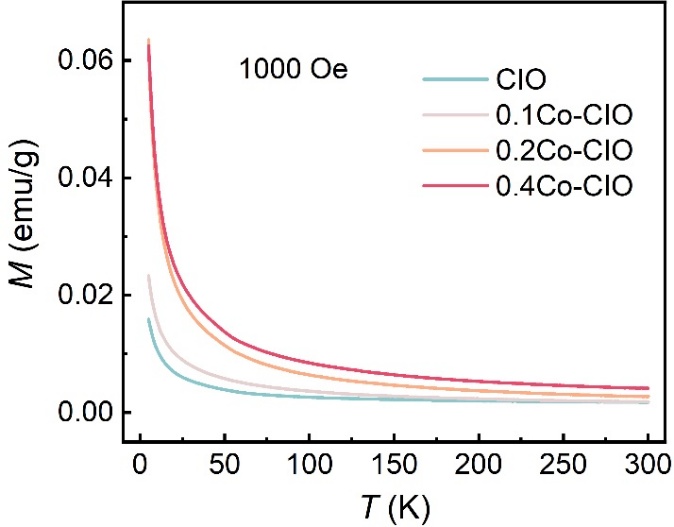
**

**Fig. S6** The Comparison of FC magnetization curve for CIO, 0.1Co-CIO, 0.2Co-CIO and 0.4Co-CIO at an applied field of 1000 Oe


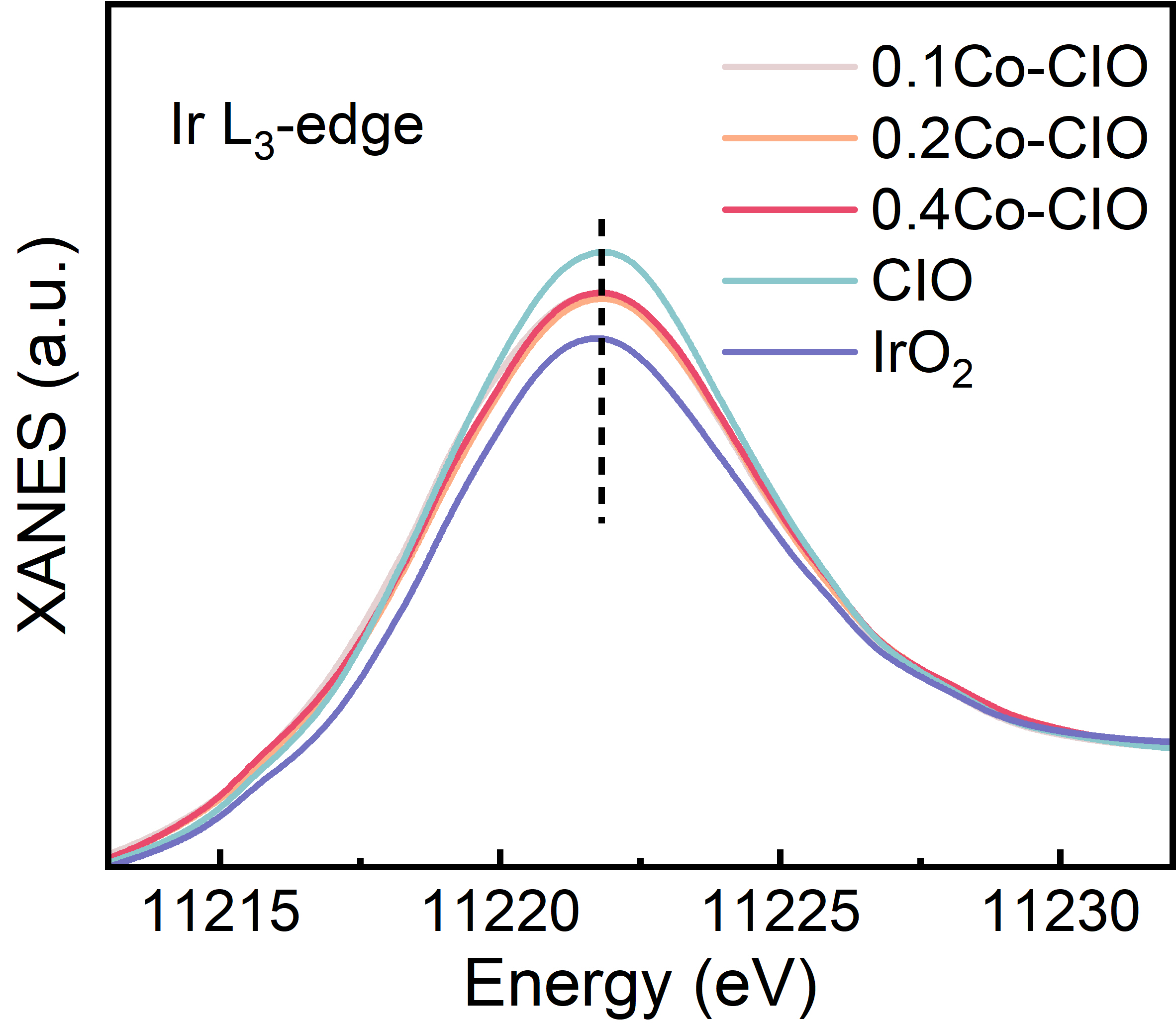


**Fig. S7** Ir *L*_3_-edge XANES region for 0.1Co-, 0.2Co-, 0.4Co-CIO, CIO and IrO_2_

**Fig. S8** **a** Co *K*-edge XANES region of 0.1Co-, 0.2Co-, 0.4Co-CIO, Co foil and Co_2_O_3_. **b** First derivatives of Co *K*-edge XANES spectra

**Fig. S9** **a** Co 2*p* XPS spectra of 0.1Co-, 0.2Co-, 0.4Co-CIO. **b** Ir 4*f* XPS spectra of 0.1Co-, 0.2Co-, 0.4Co-CIO and CIO


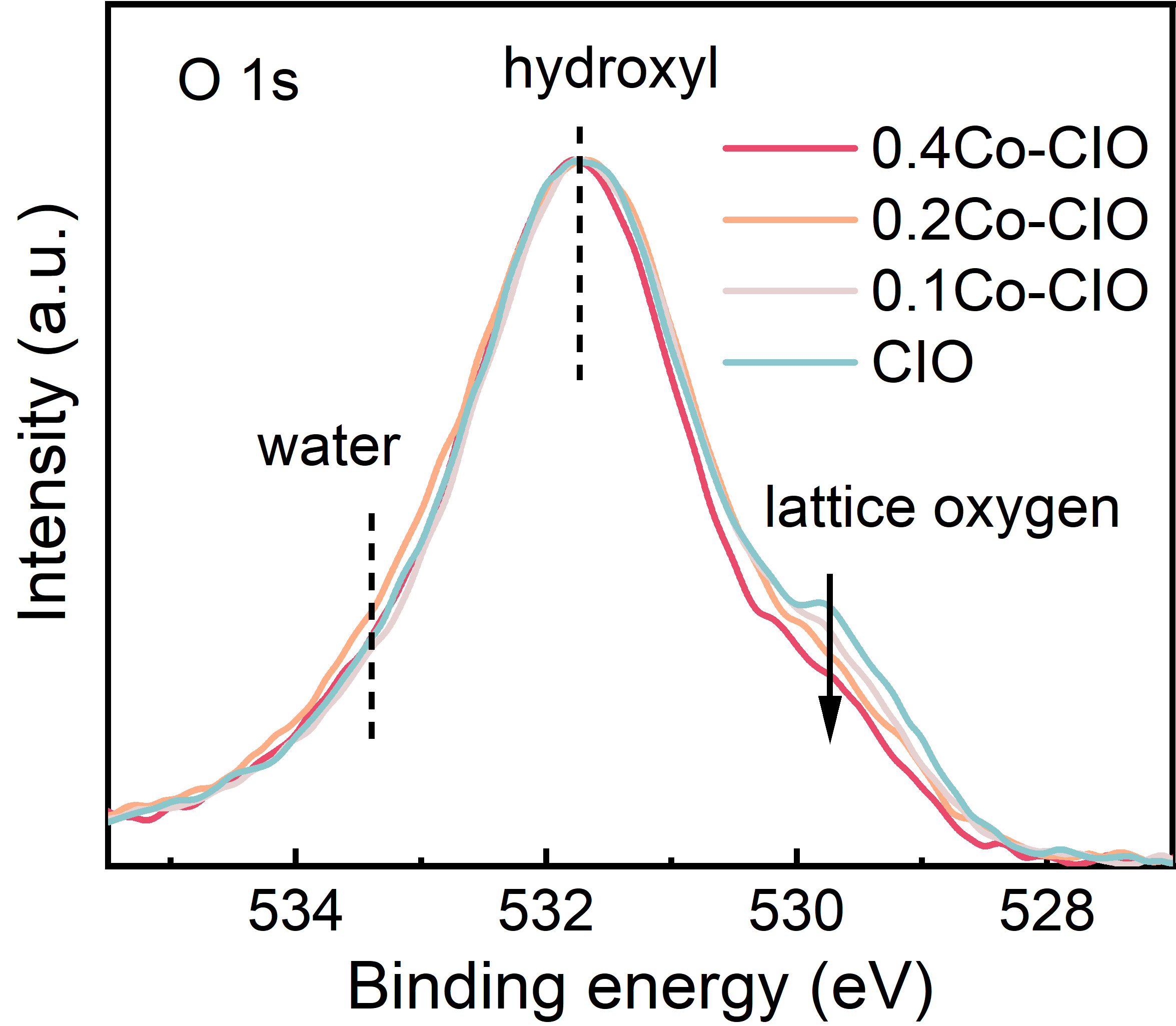


**Fig. S10** O 1*s* XPS spectra of 0.1Co-, 0.2Co-, 0.4Co-CIO and CIO


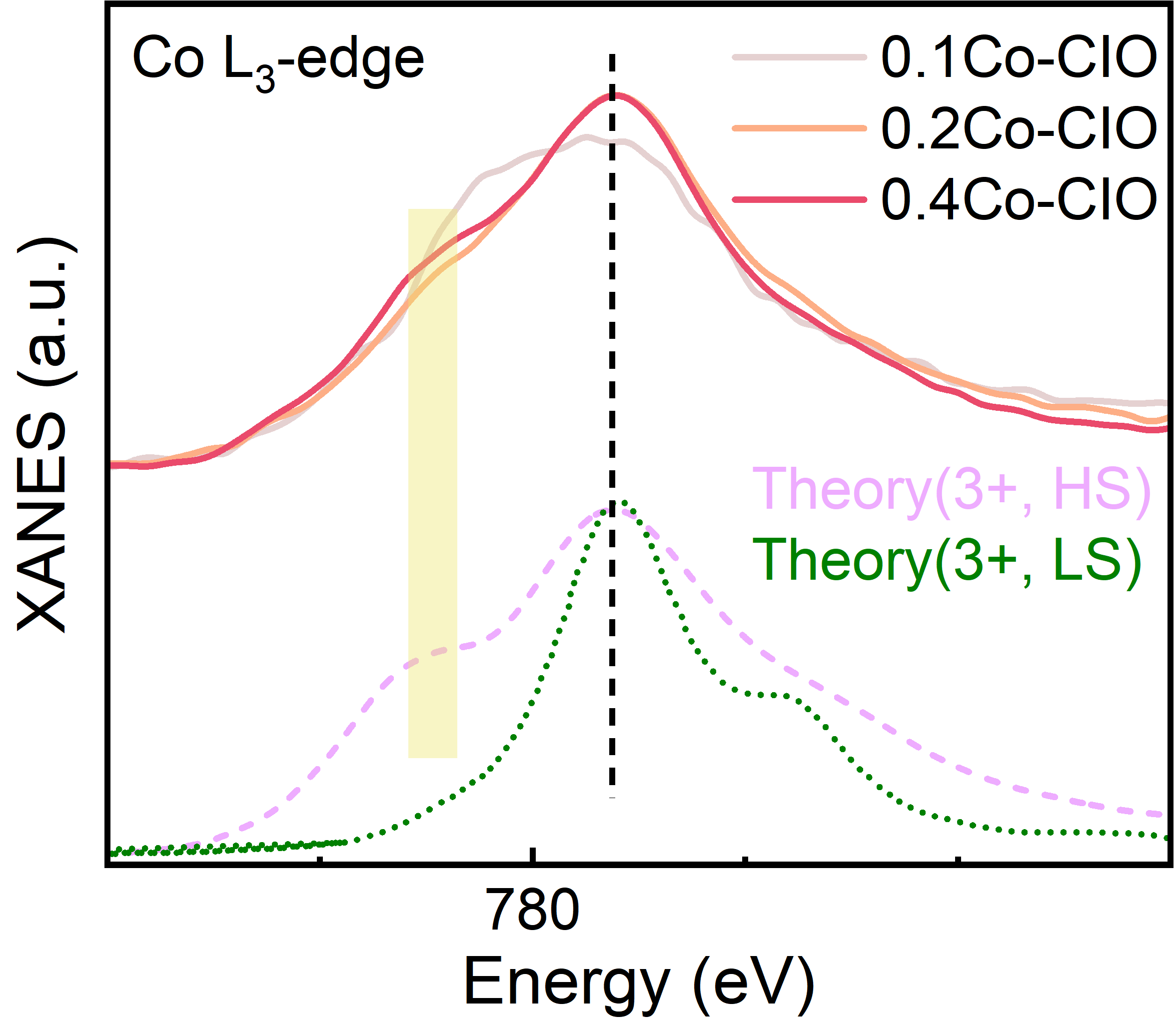


**Fig. S11** Comparison of Co *L_3_*-edge XAS spectra between Co-CIO and simulation for a Co^3+^ ion with LS and HS state in octahedral coordination

**Fig. S12** Intensity of the t_2g_ and e_g_ peaks of **a** LS state and **b** IS state Ir^4+^ due to the exchange splitting, degeneracy, the number of overlaps, and the overlap strengths

In the case of LS state Ir^4+^, there are one vacancy t_2g_ state and four vacancy e_g_ states. Besides, the t_2g_ state points in between the O atoms, and the eg states point directly to the O atoms and have σ overlap with the O 2*p* states. Effectively, this yields a difference in the hopping terms of 2:1. Additionally, the π bonding overlap with two O 2*p* orbitals and σ overlap with one O 2*p* orbital, which implies a difference in hybridization of 1:4 for each O 2*p*−Ir 5*d* overlap. The total (relative) intensity ratio for the t_2g_/e_g_ band is 1/4 × 2/1 × 1/4 = 2/16. Therefore, the expected t_2g_/e_g_ band ratio is 0.125 for LS state Ir^4+^. Similarly, the expected t_2g_/e_g_ band ratio in IS state Ir^4+^ is 2/3 × 2/1 × 1/4 = 4/12.


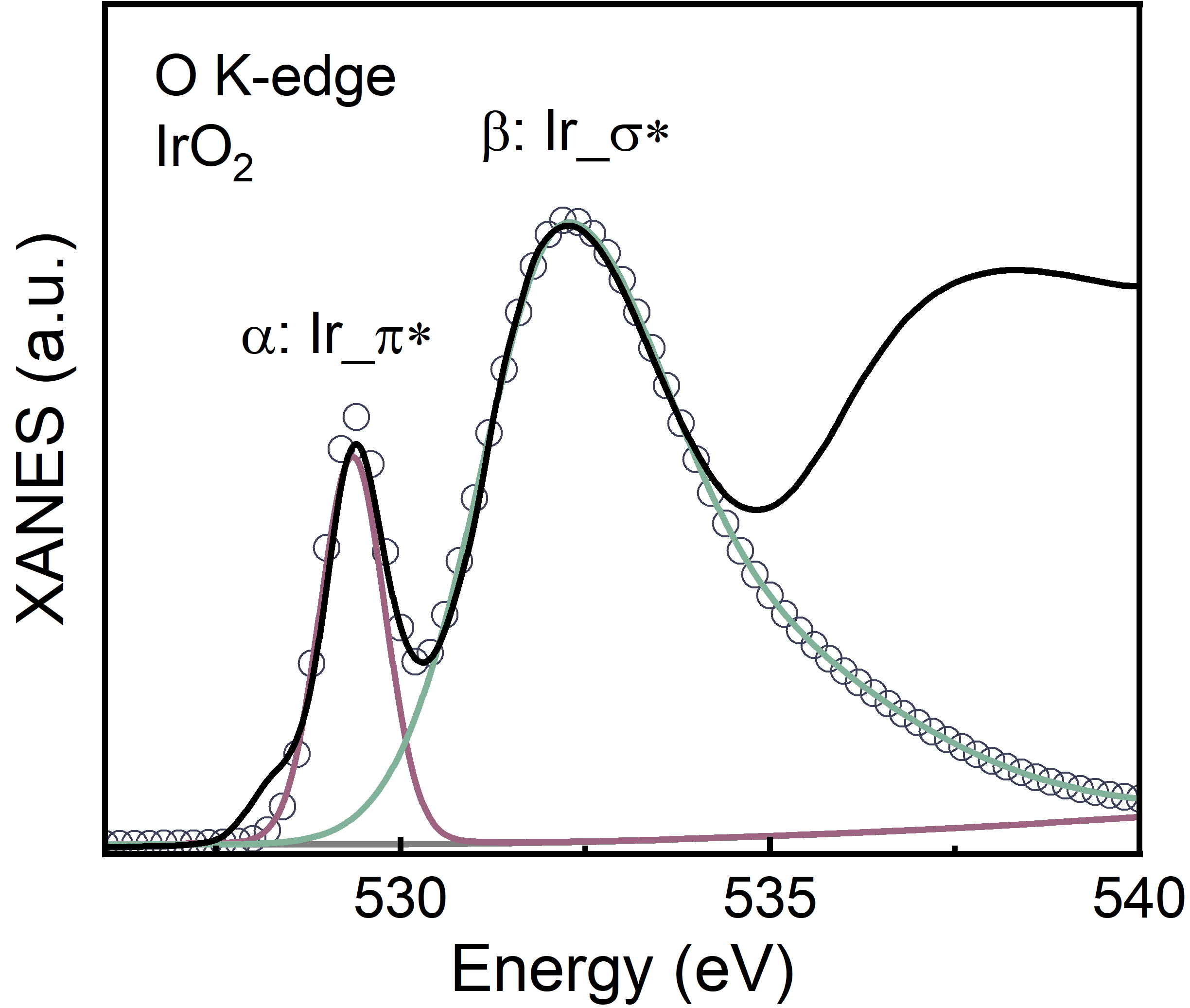


**Fig. S13** The fitting results of peak α and β in the O *K*-edge XANES for commercial IrO_2_

**Fig. S14** The fitting results of peak α and β in the O *K*-edge XANES for **a** CIO, **b** 0.1Co-CIO, **c** 0.2Co-CIO, and **d** 0.4Co-CIO

The detailed fitting results with 0.126 for IrO_2_ and 0.123 for CIO are close to the theoretical value of 0.125, validating the expected estimation of Ir spin state through the I_t2g_/I_eg_ ratio method although the fit parameters are somewhat different due to the different crystal symmetry.


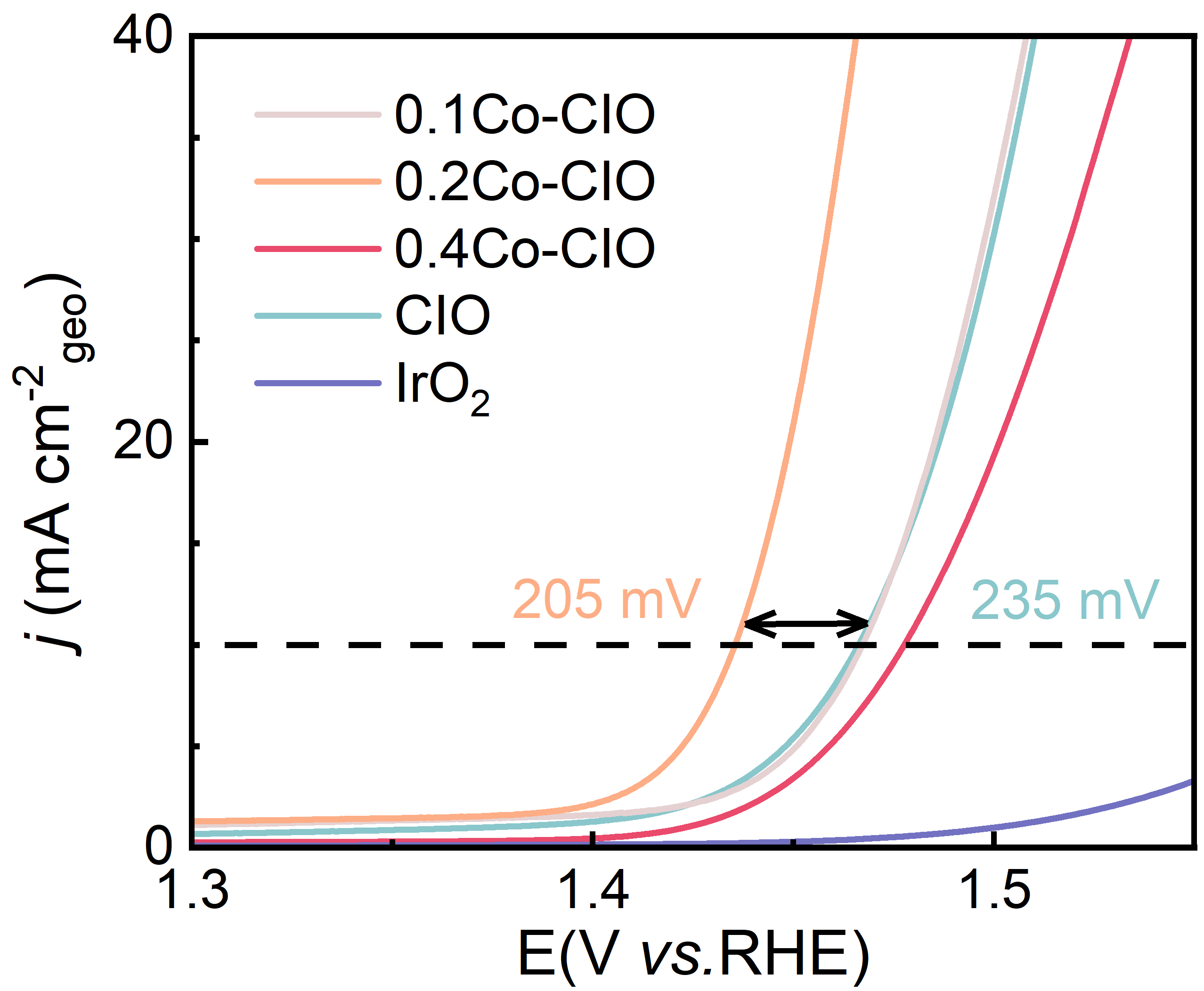


**Fig. S15** LSV polarization curves

**Fig. S16** Double-layer capacitance measurements. CVs were conducted in a non-Faradaic region of voltammogram at the following scan rate: 2, 4, 6, 8, 10 mV s^-1^. The difference in charging currents variation at an underpotential plotted against scan rate for estimation of double-layer capacitance (C_dl_)


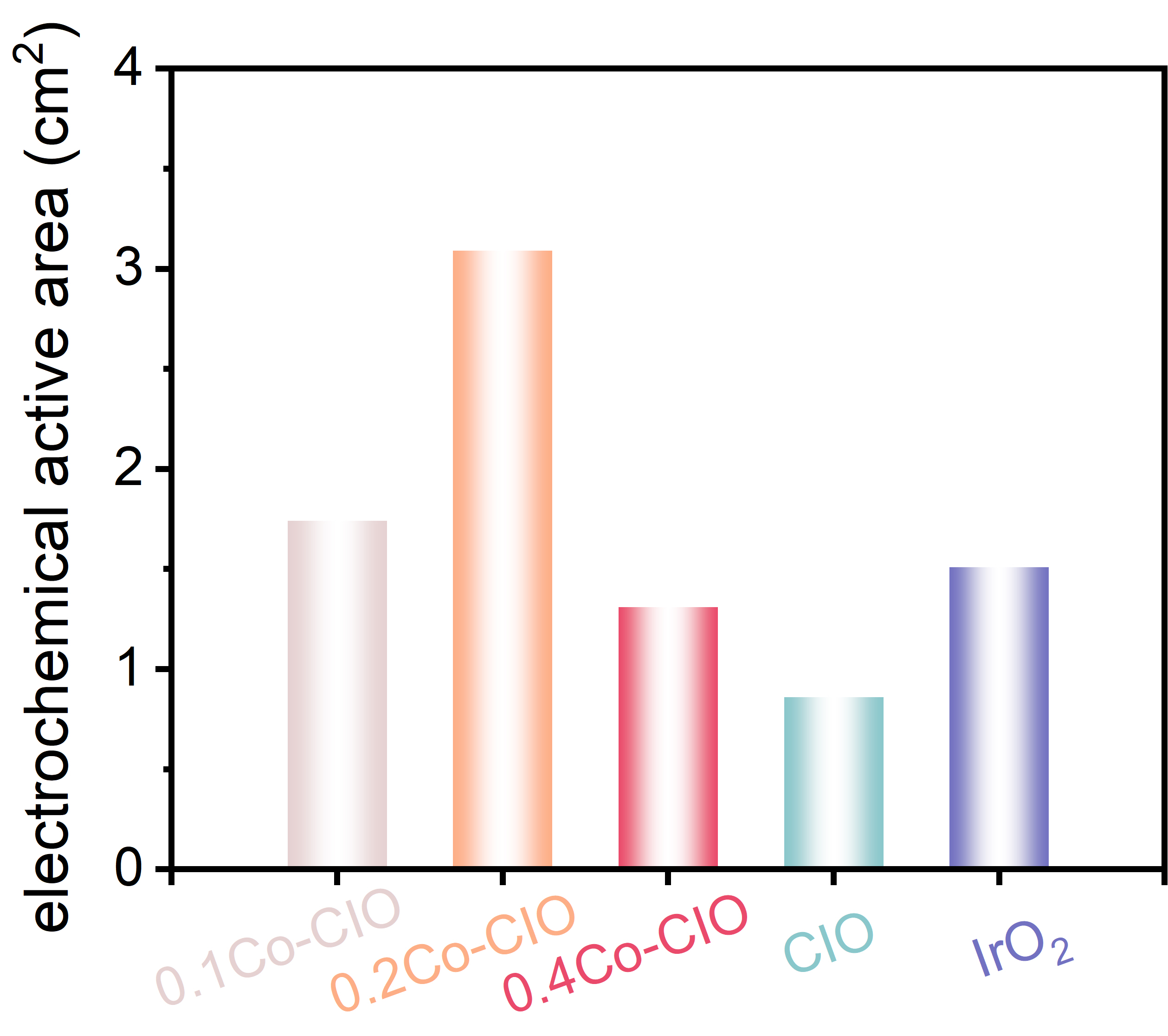


**Fig. S17** Comparison of ECSA


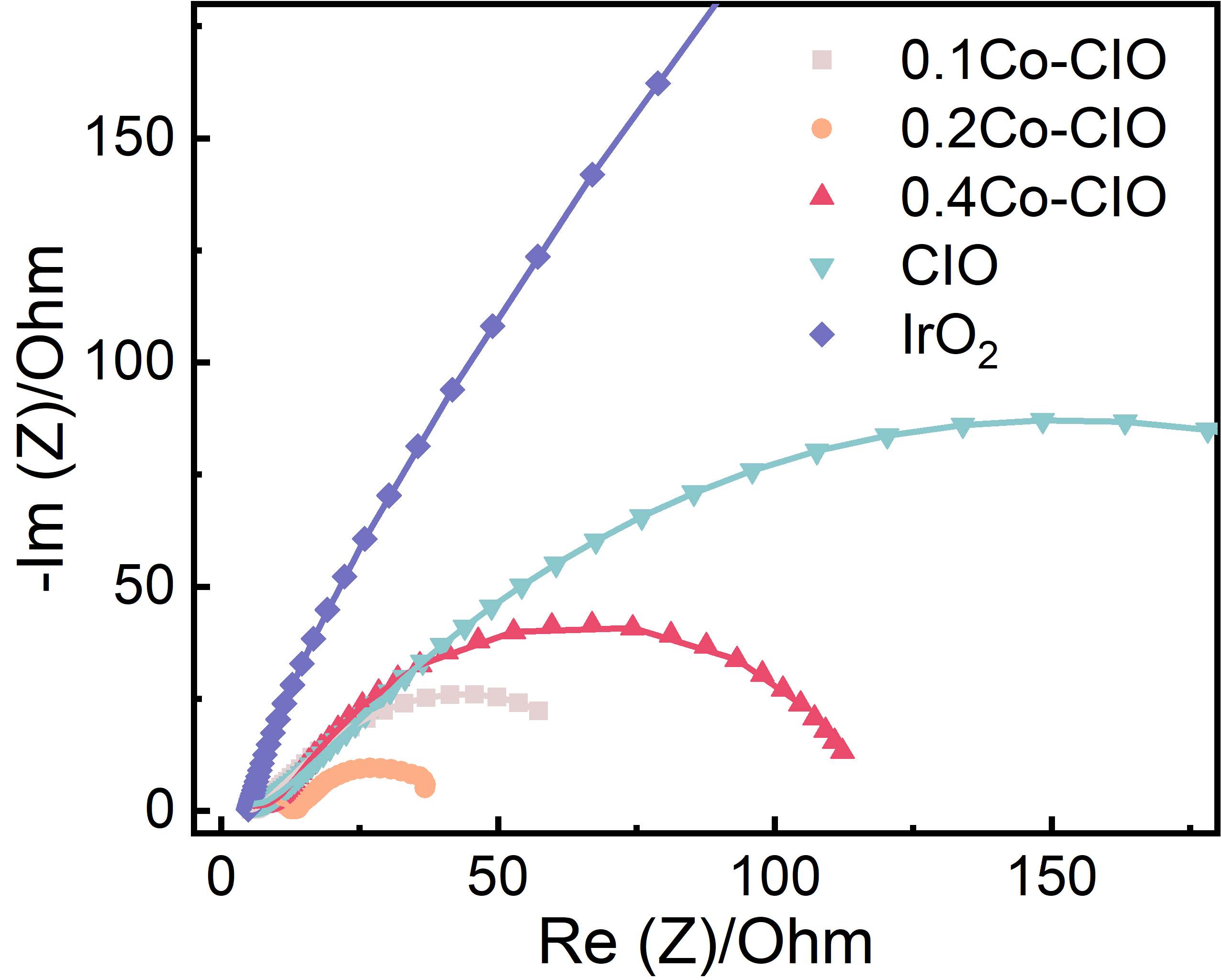


**Fig. S18** Electrochemical impedance spectra at 1.53 V (set potential)


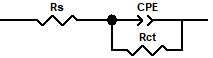


**Fig. S19** The equivalent circuit. R_s_: series resistance; R_ct_: charge-transfer resistance; CPE: the constant phase element


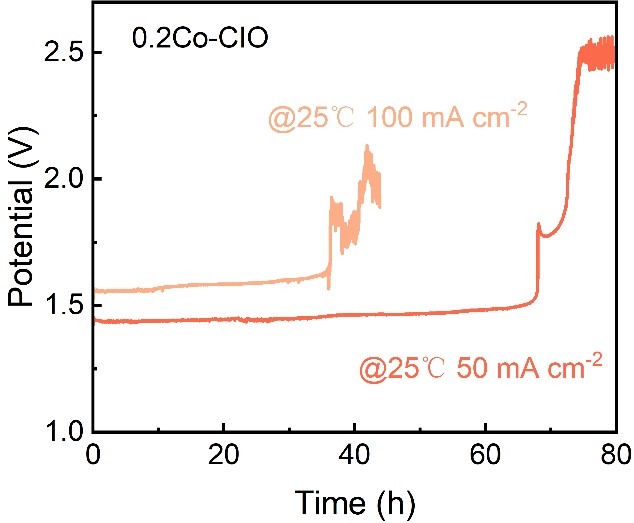
.

**Fig. S20** Chronopotentiometry curves of 0.2Co-CIO at a constant current density of 50 and 100 mA cm^-2^ (with IR compensations)

The instantaneous deactivation may be related to the fact that surface charge accumulation makes a large local potential to drive irreversible structural reorganization.

**Fig. S21** Elemental leaching was assessed by ICP-OES after cycled voltage tests of **a** 0.2Co-CIO and **b** CIO after 20, 100, 500, 2000 cycles

**Fig. S22** EDS linear scan analysis of 0.2Co-CIO (scale bar is 20 nm) as pristine and after 2000 CV cycles

**Fig. S23** TEM images of 0.2Co-CIO as **a** activated after 20 CV cycles and **b** aged after 2000 CV cycles

**Fig. S24** Comparisons of XANES at **a** Co L edge, and **b** O K edge for 0.2Co-CIO as pristine, as activated after 20 CV cycles and as aged by 2000 CV cycles


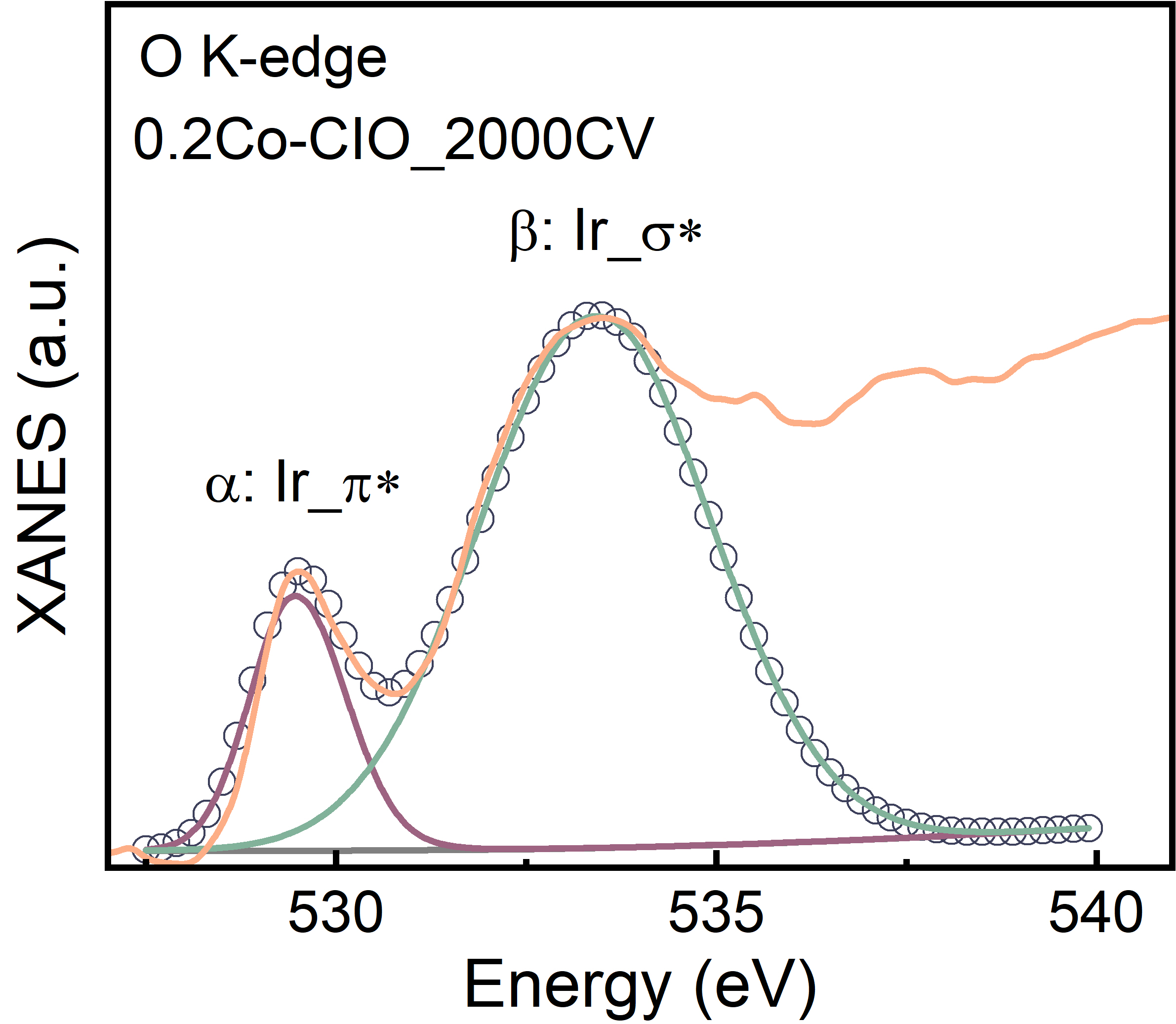


**Fig. S25** The fitting results of peak α and β in the O *K*-edge XANES for 0.2Co-CIO after aged by 2000 CV cycles


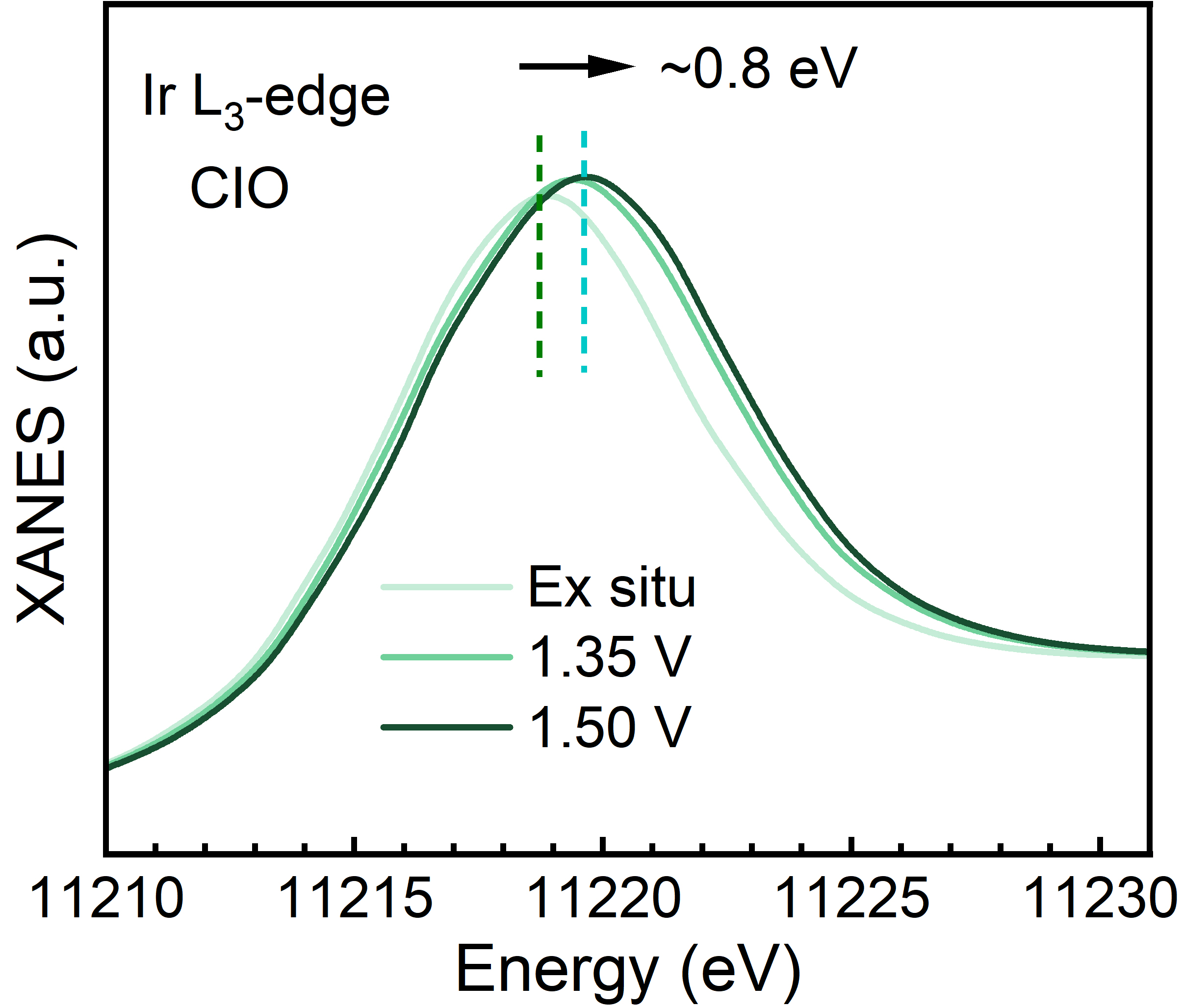


**Fig. S26** The first derivative of absorption in the XANES region of Co *K*-edge for CIO during OER process

**Fig. S27** **a** The first derivative of absorption in the XANES region of Ir *L_3_*-edge during OER process. **b** Ir *L_3_* white line positions of 0.2Co-CIO at different potentials as a function of oxidation state. White line position and oxidation state of IrCl_3_ and IrO_2_ are shown as references, respectively. All potentials are normalized to RHE

**Fig. S28** **a** The first derivative of absorption in the XANES region of Co *K*-edge during OER process. **b** Co *K*-edge positions of 0.2Co-CIO at different potentials as a function of oxidation state. Absorption edge position and oxidation state of Co_3_O_4_ and Co_2_O_3_ are shown as references, respectively. All potentials are normalized to RHE


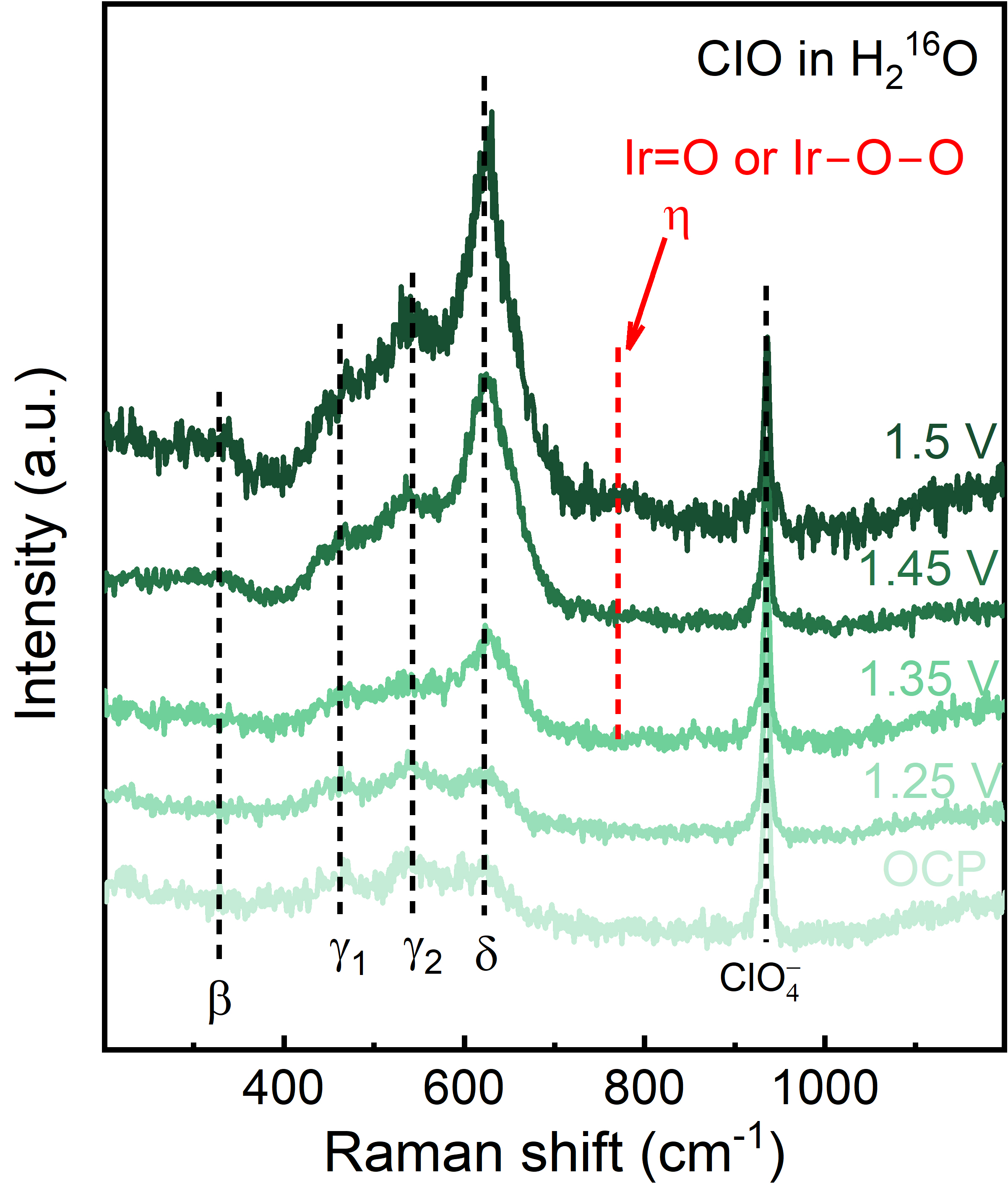


**Fig. S29** In situ Raman spectra of CIO catalyst in 1 M HClO_4_

**
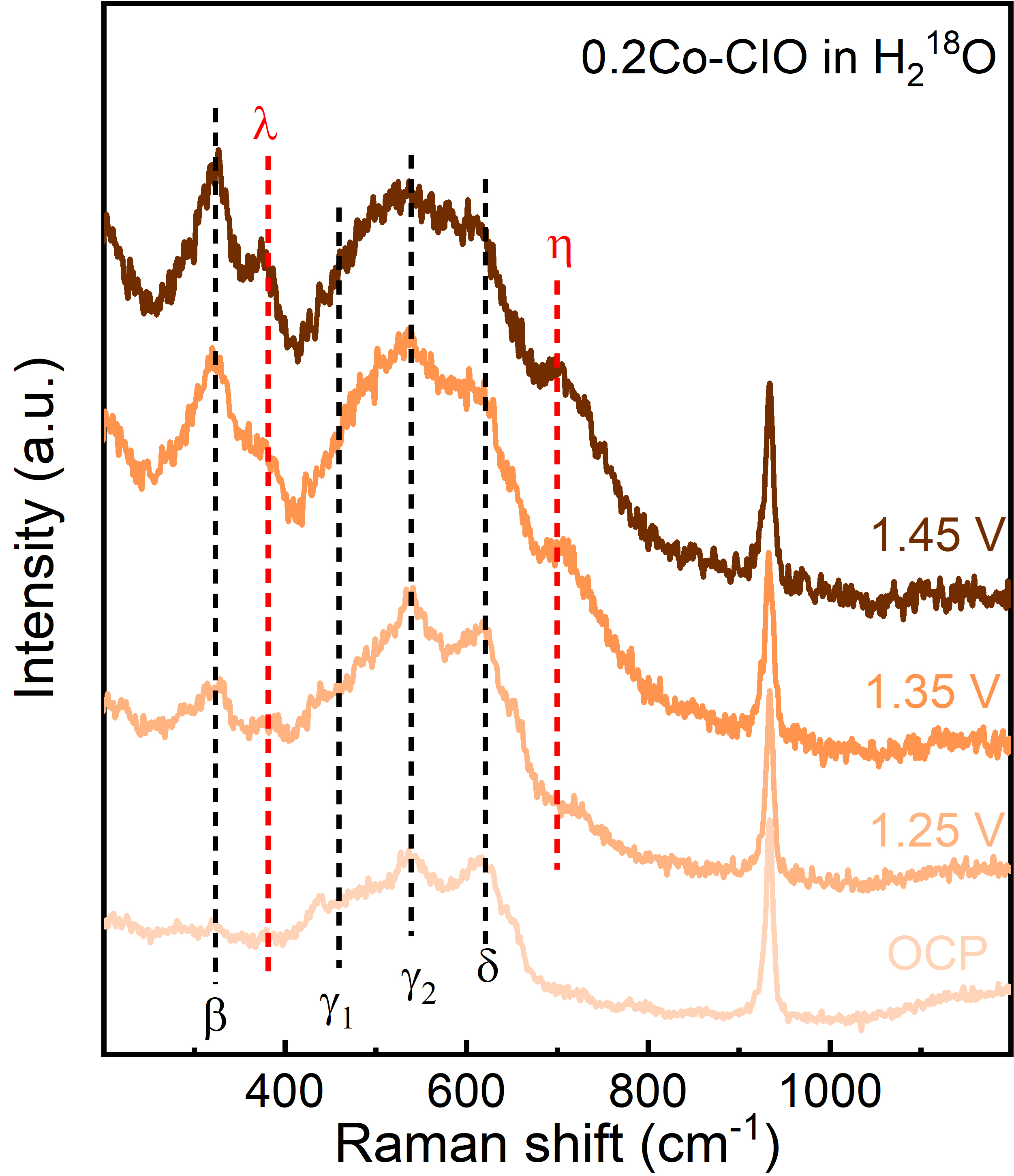
**

**Fig. S30** In situ Raman spectra of 0.2Co-CIO catalyst in 1 M HClO_4_+H_2_^18^O

**Fig. S31** DEMS signals of ^32^O_2_ (^16^O^16^O) and ^36^O_2_ (^18^O^18^O) from the reaction products for ^18^O-labeled 0.2Co-CIO and IrO_2_ catalysts in H_2_^16^O aqueous perchloric acid electrolyte and corresponding CV cycles

**Fig. S32** Slab models of **a** CIO, **b** Co1-CIO, **c** Co2-CIO, **d** Co3-CIO

DFT results indicated that Co–O–Co structure appears when Co: Ir reaches higher than that in Co3-CIO (Co: Co+Ir = 1/3), which can not effectively regulate the Ir spin state as observed for 0.4Co-CIO (Co: Co+Ir with 2/5). Therefore, higher Co concentration than that in Co3-CIO is not included.

**Fig. S33** The electron density of Co1-CIO (left) and Co2-CIO (right)

**Fig. S34** The electron spin density of Co1-CIO (left) and Co2-CIO (right)

**Fig. S35** The atomic distance of surface Ir‒Ir or Ir‒Co in **a** CIO, **b** Co1-CIO, **c** Co2-CIO, **d** Co3-CIO

The Co‒Ir atomic distance slightly reduces from 3.25 Å to 3.13 Å, 3.12 Å, and 3.07 Å as the Co amounts increase for CIO, Co1-CIO, Co2-CIO, and Co3-CIO, while the bond angle reduces from ~102° and ~108° of Ir‒O‒Ir to ~100° and ~107° of Ir‒O‒Co, respectively. The evolution of local Ir‒O‒Ir structure indicate the gradually enhanced interaction between Co and Ir atoms.


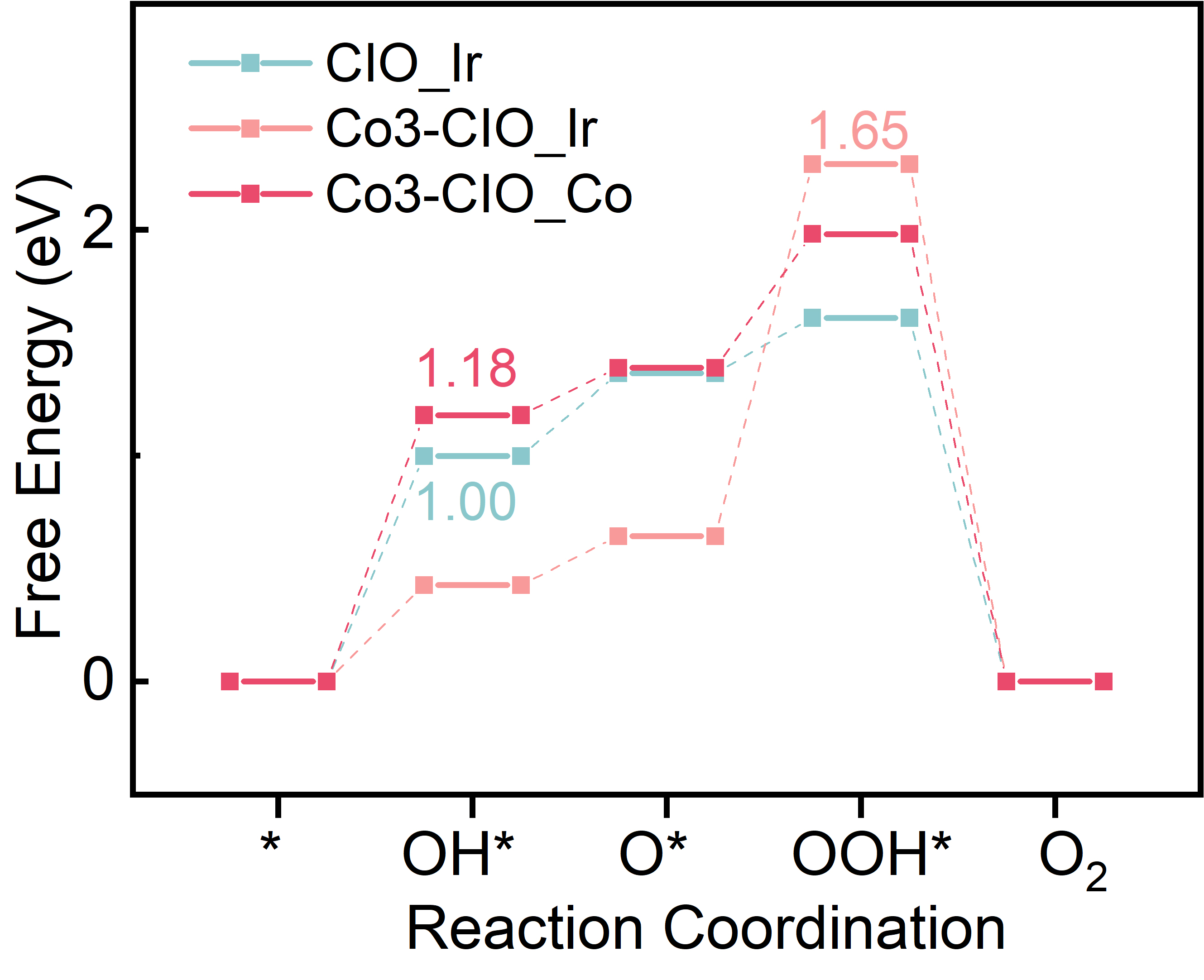


**Fig. S36** Free energy profile at 1.23 V for the AEM pathway on the Ir site of CIO and the Ir or Co site of Co3-CIO

**Table S1** Fit parameters of peak *α* and *β* in O K-edge XANES spectra

| catalysts | IrO_2_ | | | CIO | | | 0.1Co-CIO | | | | 0.2Co-CIO | | | 0.4Co-CIO | | | 0.2Co-CIO_2000CV | |
| --- | --- | --- | --- | --- | --- | --- | --- | --- | --- | --- | --- | --- | --- | --- | --- | --- | --- | --- |
| Peak | t_2g_ | e_g_ | t_2g_ | | e_g_ | t_2g_ | | e_g_ | | t_2g_ | | e_g_ | t_2g_ | | e_g_ | t_2g_ | | e_g_ |
| Position | 529.4 | 533.0 | 529.5 | | 533.9 | 529.2 | | 533.5 | | 529.1 | | 532.9 | 529.0 | | 533.0 | 529.5 | | 533.4 |
| FWHM | 1.0 | 2.7 | 1.35 | | 3.6 | 1.35 | | | 3.6 | 1.35 | | 3.6 | 1.35 | | 3.6 | 1.35 | | 3.6 |
| Area | 1.02 | 8.1 | 0.95 | | 7.70 | 1.05 | | | 8.02 | 1.69 | | 7.33 | 1.56 | | 7.56 | 1.94 | | 9.02 |
| Area ratio | 0.126 | | 0.123 | | | 0.130 | | | | 0.230 | | | 0.206 | | | 0.215 | | |
| IS (vol%) | 0 | | 0 | | | 2% | | | | 50% | | | 40% | | | 44% | | |
| IS (mole fraction) | 0 | | 0 | | | 0.18 | | | | 0.40 | | | 0.24 | | | 0.35 | | |

The same energy range of ~18 eV from 522-544 eV is carefully fitted aiming at peak α and β with different position. For quantitative studies, the Touggard background was added to remove the background signals. Other parameters including FWHM 1.35 and 3.6, as well as Lorentzian/Gaussian ratio 20% and 5% keep the same for peak α and β, respectively, based on the different spectral shape.

The fitting result of O K edge XANES spectra after 2000 CV cycles indicate a slight reduction of IS Ir atom from pristine 0.4 mole to 0.35 mole, probably due to the dissolution of surface Co atoms in acid. These similar IS Ir ratio before and after the durability test confirmed the relatively stability of Janus Co−Ir structure on surface for the long durability under 10 mA cm^‒2^ of 0.2Co-CIO for 100 h in acid.

**Table S2** Comparison of the acidic OER performances of 0.2Co-CIO with other state-of-the-art Ir-based electrocatalyst improved by transition metal doping reported recently

| catalysts | electrolytes | E/V@10  mA cm^-2^_geo_ | j (A g^-1^) | Refs. |
| --- | --- | --- | --- | --- |
| 0.2Co-CIO | 1 M HClO_4_ | 1.435 | 73@1.525 V | This work |
| Commercial IrO_2_ | 1 M HClO_4_ | 1.616 | 8 @ 1.53 V | This work |
| Sr_2_CoIrO_6_ | 0.1 M HClO_4_ | 1.535 | 73 @ 1.525 V | [S1] |
| Sr_2_NiIrO_6_ | 0.1 M HClO_4_ | 1.525 | 115 @ 1.525 V | [S2] |
| Sr_2_Co_0.9_Ir_0.1_O_3-δ_ | 0.1 M HClO_4_ | 1.55 | ———— | [S3] |
| SrIr_0.8_Zn_0.2_O_3_ | 0.1 M HClO_4_ | 1.55 | 110 @ 1.550 V | [S4] |
| Sr_2_ZnIrO_6_ | 0.1 M HClO_4_ | 1.48 | 139 @1.525 V | [S2] |
| IrCo_ae | 0.5 M H_2_SO_4_ | 1.513 | 139 @1.575 V | [S5] |
| 5Ir-Co_3_O_4_-bilayer | 0.1 M HClO_4_ | 1.54 | 1056.2 @1.54 V | [S6] |
| IrO_x_/V_O_-TiO_2_ | 0.5 M H_2_SO_4_ | 1.48 | 120.21 @1.55 V | [S7] |
| IrNiO_x_-575 | 0.05 M H_2_SO_4_ | 1.555 | 100 @1.535 V | [S8] |
| dh-IrO_X_-230 | 0.1 M HClO_4_ | 1.51 | 72 @1.51 V | [S9] |
| IrO_X_/TiO_x_ | 0.5 M H_2_SO_4_ | 1.463 | 730.8 @ 1.53 V | [S10] |
| T-0.24Ni/IrO_2_ | 0.1 M HClO_4_ | 1.42 | 1110.5 @1.58 V | [S11] |

**Table S3** Parameters resolved from Nyquist Plots

| Sample | R_s_/Ω | R_ct_/Ω |
| --- | --- | --- |
| 0.1Co-CIO | 8.954 | 72 |
| 0.2Co-CIO | 13.9 | 27.04 |
| 0.4Co-CIO | 10.55 | 111.7 |
| CIO | 11.28 | 293.4 |
| IrO_2_ | 4.385 | 4629 |

**Supplementary References**

1. R. Zhang, N. Dubouis, M. Ben Osman, W. Yin, M.T. Sougrati et al., A dissolution/precipitation equilibrium on the surface of iridium-based perovskites controls their activity as oxygen evolution reaction catalysts in acidic media. Angew. Chem. Int. Ed. **58**(14), 4571–4575 (2019). <https://doi.org/10.1002/anie.201814075>
2. M. Retuerto, L. Pascual, O. Piqué, P. Kayser, M. Abdel Salam et al., How oxidation state and lattice distortion influence the oxygen evolution activity in acid of iridium double perovskites. J. Mater. Chem. A **9**(5), 2980–2990 (2021). <https://doi.org/10.1039/d0ta10316k>
3. Y. Chen, H. Li, J. Wang, Y. Du, S. Xi et al., Exceptionally active iridium evolved from a pseudo-cubic perovskite for oxygen evolution in acid. Nat. Commun. **10**, 572 (2019). <https://doi.org/10.1038/s41467-019-08532-3>
4. M. Retuerto, L. Pascual, J. Torrero, M. Abdel Salam, Á. Tolosana-Moranchel et al., Highly active and stable OER electrocatalysts derived from Sr_2_MIrO_6_ for proton exchange membrane water electrolyzers. Nat. Commun. **13**, 7935 (2022). <https://doi.org/10.1038/s41467-022-35631-5>
5. M.F. Labata, N. Kakati, G. Li, V. Altoe, P.A. Chuang, Exploring the structure–function relationship in iridium–cobalt oxide catalyst for oxygen evolution reaction across different electrolyte media. ACS Catal. **15**(3), 1715–1726 (2025). <https://doi.org/10.1021/acscatal.4c06814>
6. G. Li, A. Priyadarsini, Z. Xie, S. Kang, Y. Liu et al., Achieving higher activity of acidic oxygen evolution reaction using an atomically thin layer of IrO*_x_* over Co_3_O_4_. J. Am. Chem. Soc. **147**(8), 7008–7016 (2025). <https://doi.org/10.1021/jacs.4c17915>
7. Q. Chu, Y. Niu, H. Tao, H. Liu, Q. Li et al., Optimizing the electronic structure of IrO*_x_* sub-2 nm clusters *via* tunable metal support interaction for acidic oxygen evolution reaction. ACS Catal. **15**(3), 1942–1951 (2025). <https://doi.org/10.1021/acscatal.4c06411>
8. S.S. Jeon, H. Jeon, J. Lee, R. Haaring, W. Lee et al., Highly active and durable iridium nickel oxide platelets for a proton exchange membrane water electrolyzer with low iridium loading. ACS Catal. **15**(6), 4963–4974 (2025). <https://doi.org/10.1021/acscatal.5c00014>
9. K. Sun, X. Liang, X. Wang, Y.A. Wu, S. Jana et al., Highly efficient and durable anode catalyst layer constructed with deformable hollow IrO_x_ nanospheres in low-iridium PEM water electrolyzer. Angew. Chem. **137**(21), e202504531 (2025). <https://doi.org/10.1002/ange.202504531>
10. Y. Qin, Y. Huang, Q. Ye, J. Wang, M. Endo et al., In situ construction of IrO_x_ Nanofilm on TiOx for boosting low-Ir catalysis in practical PEM electrolyze. Adv. Energy Mater. **15**(21), 2405636 (2025). <https://doi.org/10.1002/aenm.202405636>
11. Liu, X. Zhong, X. Chen, D. Wu, C. Yang et al., Unraveling compressive strain and oxygen vacancy effect of iridium oxide for proton-exchange membrane water electrolyzers. Adv. Mater. **37**(16), 2501179 (2025). <https://doi.org/10.1002/adma.202501179>
